# Socioeconomic position as a predictor of youth's movement trajectory profiles between ages 10 and 14 years

**DOI:** 10.1186/s12966-023-01491-5

**Published:** 2023-07-22

**Authors:** Katrina Wilhite, Borja del Pozo Cruz, Michael Noetel, Chris Lonsdale, Nicola D. Ridgers, Carol Maher, Emma Bradshaw, Taren Sanders

**Affiliations:** 1 Institute for Positive Psychology and Education, Australian Catholic University, 33 Berry Street, Sydney, NSW 2060 Australia; 2 Center for Active and Healthy Ageing, Department of Sport Sciences and Clinical Biomechanics, University of Southern Denmark, Odense, Denmark; 3 School of Behavioural and Health Sciences, Australian Catholic University, Brisbane, Australia; 4 Alliance for Research in Exercise, Nutrition and Activity, University of South Australia, Adelaide, Australia

**Keywords:** Physical activity, Sedentary behavior, Sleep, Children, Adolescents, Socioeconomic position

## Abstract

**Background:**

Combinations of movement behaviors (i.e., physical activity, sedentary behavior, sleep) are associated with health and developmental outcomes in youth. Youth vary in how they accumulate these behaviors, both in volume and specific domains (e.g., sedentary time spent on recreational screen activities vs homework). The aim of this study was to examine how youth’s combined general and domain-specific movement trajectories differ by socioeconomic position.

**Methods:**

We conducted a longitudinal, group-based multi-trajectory analysis to identify general and domain-specific movement trajectory profiles for 2457 youth from age 10 to 14 years from the Longitudinal Study of Australian Children from 2014–2018. We used multinomial logistic regression to test if socioeconomic position predicted profile membership.

**Results:**

We identified three general movement trajectory profiles for both sexes, four domain-specific profiles for males, and five for females. For general movement trajectories, females from lower socioeconomic positions were more likely to be a combination of less active and more sedentary than females from higher socioeconomic positions. Males across socioeconomic positions spend similar amounts of time in physical activity, sedentary time, and sleep. For domain-specific movement trajectories, youth from lower socioeconomic positions were likely to spend a combination of less time in education-based sedentary behavior and more time in recreational screen activities than their higher socioeconomic position peers.

**Conclusion:**

Our results indicate that socioeconomic position predicted in which domains youth accumulate their movements. Future observational research and interventions targeting different socioeconomic groups should therefore consider domain-specific movement trajectories.

**Supplementary Information:**

The online version contains supplementary material available at 10.1186/s12966-023-01491-5.

## Introduction

The effects of movement behaviors (i.e., physical activity, sedentary behavior, and sleep) on youth’s outcomes have been widely researched [1–3]. Recently, several countries and the World Health Organization have updated their health recommendations to combine all movement behaviors in a 24-h time frame, instead of individually [4, 5]. Time spent in one movement behavior displaces time spent in others [6]. Consequently, there is likely an association between 24-h movement profiles (i.e., a combination of all movement behaviors) and how they influence youth's physical, psychological, and educational outcomes [6–8]. Isotemporal substitution and compositional data analysis are methods used to better understand the interactions of movement behaviors with outcomes [9, 10]. These methods address the limitation of “combining” individually measured movement behaviors to create a “day” that may add up to be greater than or less than 24 h. However, isotemporal substitution and compositional data analysis only provide estimations, and many studies are cross-sectional [11, 12]. Therefore, other methods using longitudinal data are additionally needed.

Trajectories in youth’s movement behaviors, particularly from primary to secondary school, should be investigated because this time period presents many factors (e.g., school setting, social life, biological and cognitive maturation) that may influence a change in movement behaviors [13–15]. Understanding youth’s movement trajectory profiles may help identify those at risk of developing poor health, psychological, or academic outcomes during the primary to secondary school transition. However, two systematic reviews have revealed the lack of longitudinal studies that include all three movement behaviors [8, 16]. One review particularly investigated the changes in combinations of movement behaviors from primary to secondary school but found no studies including all three movement behaviors during this critical time period [16]. Therefore, longitudinal research in this age group is warranted.

Domain-specific movement behaviors should also be considered because the type of behavior (e.g., educational sedentary behavior versus recreational screen activities) may influence their associations with outcomes in important ways. For example, a meta-analysis found that when compared with household physical activity, leisure-time physical activity and active transportation were more positively associated with mental health [17]. Sedentary time spent reading benefits cognitive development [18], but high volumes of sedentary screen time may be associated with increased depressive symptoms, higher adiposity, and lower quality of life [19, 20]. Studies show that insufficient nighttime sleep duration leads to increased odds of obesity, metabolic dysfunction, and can be detrimental to youth's academic success, motivation, and attention [21–24]. However, some studies suggest that daytime naps, independent of nighttime sleep, benefit youth’s neurocognitive function, psychological wellness, behavior problems, and academic achievement [25, 26]. Therefore, it is important to include domain-specific sleep (i.e., naps vs nighttime sleep) in sleep analyses and further explore its effects on youth. Ultimately, the direction and magnitude of an association with an outcome may differ depending on the explored domain-specific movement behavior.

In high-income countries, when total physical activity is studied in isolation, research shows that youth from families of lower socioeconomic positions are less physically active than youth from higher socioeconomic positions [27]. Studies show contradictory evidence on whether youth from higher or lower socioeconomic positions spend more time in sedentary behavior [28, 29]. Youth from lower socioeconomic positions have reported poorer sleep [30]. However, these conclusions were based mainly on cross-sectional studies, and none examined combinations of movement behaviors. Whether there is an association between socioeconomic position and movement profiles in youth is, therefore, unknown [16].

Also unknown is the association between socioeconomic position and youth’s domain-specific movement behaviors. When measuring movement behaviors in isolation, youth from higher socioeconomic positions participate in more organized sports [31] while those from lower socioeconomic positions participate in more unstructured physical activity and active transportation [32, 33]. Youth from lower socioeconomic positions tend to engage in more screen time compared to those from higher socioeconomic positions [29, 34], while those from higher socioeconomic positions tend to spend more time reading and playing music than youth from lower socioeconomic positions [19, 35]. Youth from lower socioeconomic positions have longer nap durations than those from higher socioeconomic positions [36].

It is unclear how youth’s movement changes over time and whether those from different socioeconomic positions differ in how they spend their 24-h day regarding movement. Understanding the impact of socioeconomic position on youth’s movement trajectories may identify areas to intervene and inform future guidelines (e.g., reducing sedentary behavior in schools [37]) to help those most in need. Several countries and the World Health Organization have identified health disparities across socioeconomic positions as a major problem that must be addressed [38–40]. Identifying differences in health behaviors, such as how youth move, may be the first step in making changes toward decreasing health inequities. Therefore, this study aims to answer four questions:

1. What are the different general movement trajectory profiles regarding the overall, combined quantity of physical activity, sedentary behavior, and sleep among youth in Australia?
2. What are the combined domain-specific movement trajectory profiles among youth in Australia?
3. Does socioeconomic position predict profile membership in youth’s general movement trajectory profiles regarding overall physical activity, sedentary behavior, and sleep?
4. Does socioeconomic position predict profile membership in youth’s domain-specific movement trajectory profiles?

## Methods

### Dataset

We analyzed data from *The Longitudinal Study of Australian Children* (LSAC) [41]. Data has been collected on two nationally representative cohorts of children every two years since 2004. We used data from the three most recent waves (Waves 6–8, 2014–2018) of the younger cohort when the participants were aged 10–11, 12–13, and 14–15 years. Data from earlier waves were not included due to a change in data collection procedures in the time-use diaries (e.g., updated coding categories, free time responses replaced 15-min increments) which could not be harmonized [42].

### Time-use

Each participant completed a paper time-use diary for one day including the start time of each activity, who accompanied them, where they were, and any concurrent activities they performed [42]. Time-use diaries were reviewed through an interview the next day. We calculated the time each participant spent in a given activity by subtracting the start time of an activity from the start time of the following activity. For the day's final activity, we subtracted the last activity’s start time from the participant’s “sleep time”. Participants filled out their time-use diary on the same day of the week at each timepoint [42].

We assigned the pre-coded time-use diary activities general and domain-specific movement behaviors (see Table 1). A list of coded activities can be found in Additional file 1. Domain-specific categories were based on categories defined by previous studies [17, 43, 44]. In cases where concurrent activities were recorded, typically the main activity was coded. In instances where it was likely that a concurrent activity took precedence regarding movement (e.g., the participant was likely sedentary rather than active), the concurrent activity was coded. For example, if the main activity “babysitting'' was paired with “watching television”, the activity would be assigned to the domain-specific movement behavior of “recreational screen time” since the participant was sedentary.

**Table 1:** Movement behavior categories

General movement behaviors	Domain-specific movement behaviors
Light physical activity	Active transportation
	Leisure-time
	Work/Household
Moderate-vigorous physical activity	Structured
	Unstructured
Sleep	Nighttime sleep
	Daytime naps
Sedentary behavior	Education-based
	Leisure-time (non-screen based)
	Passive transportation
	Recreational screen activities
	Self-care
	Social-based

To be included in the study, participants had to have (a) a valid time-use diary at Wave 6 (i.e., no missing information, no misordered events), (b) recorded their sleep and wake times, and (c) included their socioeconomic position data. Outliers were excluded if the time-use diary entries appeared to be an atypical weekday (e.g., passive transportation >  = 8 h, self-care >  = 4 h). Decision rules for outliers made by the researchers can be found in Additional file 2. A sensitivity analysis was run by conducting the analysis with the outliers included.

We handled missing follow-up time-use diary data through multiple imputation with the mice package, version 3.14.0, in R [45]. Variables in the imputation included time spent in each movement behavior, sex, age, Indigenous status (i.e., Aboriginal, Torres Strait Islander, or not Aboriginal), remoteness (ranging from 1 [Highly Accessible]—5 [Very Remote]), socioeconomic position, and day of the week the diary was filled out. We used five imputed datasets. There were 25.7% of participants missing time-use diary data at Wave 7, 36.7% at Wave 8, and 17.5% missing time-use diary data across both waves. There was no missing data for sex, age, Indigenous status, or remoteness.

We analyzed data from male and female participants separately due to previously reported differences in their daily activities [46, 47]. Additionally, when comparing models combining males and females versus stratified models, the Bayesian information criterion indicated the stratified models had a better fit. Only weekday data was analyzed due to the limited data available for weekend participants.

### Socioeconomic position

We assessed socioeconomic position through LSAC’s socioeconomic position variable, a z-score among all families [48]. The socioeconomic position variable is a normalized variable that was developed by LSAC to rank each family relative to other families in the study. The ranking was determined by standardizing each family’s combined income, the highest education completed by each parent (coded into years of education and standardized), the occupational status of each parent (coded into categories according to the Australian and New Zealand Standard Classification of Occupations [49]), and whether the participant’s family was a single or two-parent home.

### Data analysis

We used longitudinal data from Waves 6–8 to perform group-based multi-trajectory analysis with the gbmt package in R, version 0.1, to find movement trajectory profiles between the ages of 10–14 years [50]. Group-based multi-trajectory analysis used finite mixture modeling to derive distinct groups of participants with similar trajectories in multiple variables concurrently [51]. This is done by identifying clusters of youth who are most likely to have similar combinations of movement trajectories based on the given data. Time spent in each coded activity at ages 10, 12, and 14 was entered into the model. Normalization was not necessary for this analysis since all trajectories were in the same units (minutes). The analysis was run separately to find general and domain-specific movement trajectory profiles. See Additional file 3 for gbmt code.

To determine the most appropriate number of groups for each analysis, we used the fit-criteria assessment plot tool to compare the Bayesian information criterion (BIC), average posterior probability, and odds of correct classification to compare 10 models (linear and quadratic polynomials of groups sizes between 2 – 6) [52]. The model with the lowest BIC, average posterior probability (minimum 0.70), and odds of correct classification (minimum 5.0) was chosen. If two models had similar results, we plotted both to visually determine if extra groups provided novel information.

We used the nnet package, version 7.3.16, in R [53] to run a multinomial logistic regression test to evaluate if socioeconomic position was associated with profile membership. We chose a reference group with the highest hypothesized health benefits in each analysis. Thus, we selected the group with the trajectory most similar to the following pattern: participants, in combination, increased their moderate-to-vigorous physical activity, light physical activity, and sleep, but decreased their sedentary behavior, provided the quantity of these behaviors was likely to be associated with the most desirable outcomes [8].

## Results

### Participant characteristics

In Wave 6, 3764 B-Cohort participants completed data collection. Of these, 3460 completed time-use diaries. We excluded 145 time-use diaries due to incorrect times being recorded in the diary, 26 due to missing socioeconomic position data, and six due to missing sleep data. We removed 140 participants as outliers. This resulted in 3143 participants, 2457 providing weekday data. Of these participants, 1251 were male and 1206 were female. Participant descriptive characteristics can be found in Table 2. Included participants did not differ from excluded participants in remoteness (determined by the Australian Standard Geographic Classification), sex, or Indigenous background.

**Table 2:** Summary of participant characteristics and movement behaviors

	**Male (*N* = 1251)**	**Female (*N* = 1206)**	**Overall (*N* = 2457)**
**Participant Details**			
Age	10 (± 0.49)	10 (± 0.49)	10 (± 0.49)
Indigenous			
*Not Aboriginal*	1216 (97%)	1173 (97%)	2389 (97%)
*Aboriginal*	29 (2%)	29 (2%)	58 (2%)
*Torres Strait Islander*	4 (0%)	2 (0%)	6 (0%)
*Both*	2 (0%)	2 (0%)	4 (0%)
Remoteness			
*Highly Accessible*	642 (51%)	606 (50%)	1248 (51%)
*Accessible*	349 (28%)	357 (30%)	706 (29%)
*Moderately Accessible*	217 (17%)	199 (17%)	416 (17%)
*Remote*	25 (2%)	19 (2%)	44 (2%)
*Very Remote*	12 (1%)	17 (1%)	29 (1%)
*Not determined*	6 (0%)	8 (1%)	14 (1%)
Socioeconomic Position	0.034 (± 0.99)	0.022 (± 1.0)	0.028 (± 1.0)
**Domain-Specific Movement Behaviors**			
Active Transport (10)	0 (0, 10)	0 (0,10)	0 (0,10)
Active Transport (12)	0 (0,15)	0 (0,17)	0 (0,15)
Active Transport (14)	0 (0,20)	0 (0,20)	0 (0,20)
Unstructured Light Physical Activity (10)	30 (0,90)	35 (0,94)	30 (0,90)
Unstructured Light Physical Activity (12)	0 (0,44)	10 (0,70)	5 (0,65)
Unstructured Light Physical Activity (14)	0 (0,30)	0 (0,40)	0 (0,40)
Work and Household Light Physical Activity (10)	44 (20,75)	55 (30,90)	64 (25,81)
Work and Household Light Physical Activity (12)	59 (30,94)	75 (45,120)	65 (35,109)
Work and Household Light Physical Activity (14)	56 (30,96)	73 (41,115)	64 (25,105)
Unstructured Moderate-Vigorous Physical Activity (10)	0 (0,55)	0 (0,16)	0 (0,33)
Unstructured Moderate-Vigorous Physical Activity (12)	0 (0,30)	0 (0,0)	0 (0,15)
Unstructured Moderate-Vigorous Physical Activity (14)	0 (0,35)	0 (0,0)	0 (0,0)
Structured Moderate-Vigorous Physical Activity (10)	0 (0,45)	0 (0,40)	0 (0,45)
Structured Moderate-Vigorous Physical Activity (12)	0 (0,40)	0 (0,34)	0 (0,40)
Structured Moderate-Vigorous Physical Activity (14)	0 (0,34)	0 (0,36)	0 (0,49)
Nighttime Sleep (10)*	580 (± 63)	590 (± 64)	590 (± 64)
Nighttime Sleep (12)*	570 (± 78)	570 (± 82)	570 (± 80)
Nighttime Sleep (14)*	550 (± 95)	550 (± 86)	550 (± 91)
Daytime Naps (10)	0 (0,0)	0 (0,0)	0 (0,0)
Daytime Naps (12)	0 (0,0)	0 (0,0)	0 (0,0)
Daytime Naps (14)	0 (0,0)	0 (0,0)	0 (0,0)
Education Based Sedentary Behavior (10)	270 (270,334)	280 (50,345)	274 (44,340)
Education Based Sedentary Behavior (12)	238 (0,350)	227 (15,360)	235 (0,355)
Education Based Sedentary Behavior (14)	215 (0,330)	225 (0,360)	220 (0,349)
Leisure Time Sedentary Behavior (10)	120 (64,195)	140 (77,215)	130 (70,203)
Leisure Time Sedentary Behavior (12)	110 (45,195)	120 (45,198)	115 (45,195)
Leisure Time Sedentary Behavior (14)	70 (20,145)	84 (29,165)	75 (24,155)
Passive Transport (10)	40 (15,75)	50 (20,83)	45 (19,80)
Passive Transport (12)	50 (20,90)	55 (25,90)	50 (20,90)
Passive Transport (14)	45 (10,89)	54 (20,92)	50 (17,90)
Recreational Screen Activities (10)	162 (74,280)	105 (45,204)	131 (59,240)
Recreational Screen Activities (12)	184 (90,305)	163 (58,235)	151 (70,275)
Recreational Screen Activities (14)	230 (120,380)	180 (80,303)	201 (99,345)
Self-Care Sedentary Behavior (10)	10 (0,20)	10 (0,25)	10 (0,20)
Self-Care Sedentary Behavior (12)	10 (0,0)	0 (0,0)	0 (0,0)
Self-Care Sedentary Behavior (14)	0 (0,0)	0 (0,0)	0 (0,0)
Social-Based Sedentary Behavior (10)	11 (0,63)	25 (0,82)	17 (0,74)
Social-Based Sedentary Behavior (12)	20 (0,80)	50 (0,125)	35 (0,105)
Social-Based Sedentary Behavior (14)	50 (0,115)	75 (20,144)	60 (13,130)
**General Movement Behaviors**			
Light Physical Activity (10)	103 (54,173)	124 (70,194)	114 (60,183)
Light Physical Activity (12)	105 (57,170)	126 (75,196)	115 (65,185)
Light Physical Activity (14)	95 (54,161)	112 (67,180)	105 (60,170)
Moderate-Vigorous Physical Activity (10)	40 (0,100)	0 (0,69)	24 (0,90)
Moderate-Vigorous Physical Activity (12)	15 (0,90)	0 (0.70)	49 (0,80)
Moderate-Vigorous Physical Activity (14)	30 (0,105)	0 (0,60)	0 (0,90)
Sleep (10)*	590 (± 63)	590 (± 64)	590 (± 63)
Sleep (12)*	580 (± 81)	580 (± 88)	580 (± 84)
Sleep (14)*	560 (± 99)	560 (± 89)	560 (± 95)
Sedentary Behavior (10)*	660 (± 120)	650 (± 120)	650 (± 120)
Sedentary Behavior (12)*	670 (± 150)	660 (± 140)	660 (± 140)
Sedentary Behavior (14)*	690 (± 150)	700 (± 150)	690 (± 150)

Values represent median (interquartile range) or count (%); * = mean (standard deviation); Age is measured in years; Socioeconomic Position is a composite score of income, educational attainment, and occupational status. The variable is standardized so, the mean is 0 and standard deviation is 1 (range -5.529 – 2.733).; Movement Behaviors are measured in minutes; (10) = at age 10; (12) = at age 12; (14) = at age 14

### General movement trajectories

**Fig. 1:**
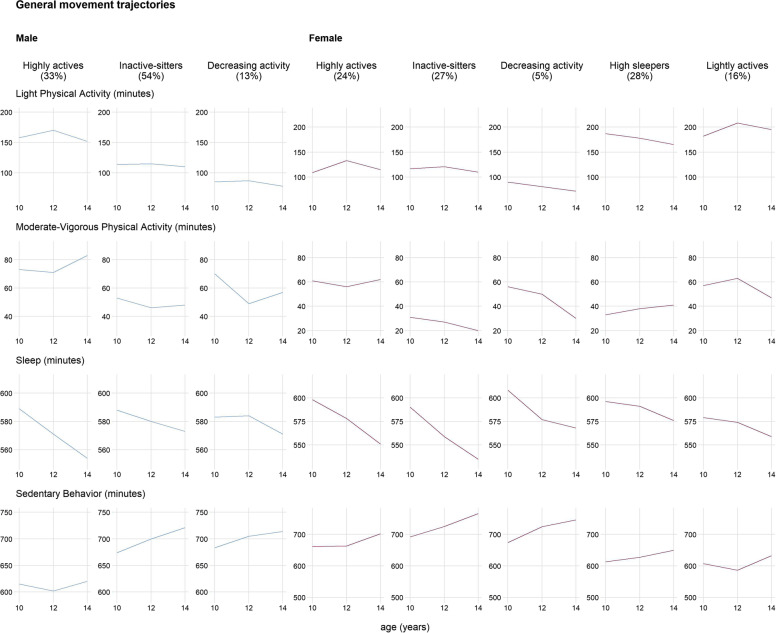
General movement trajectories

For general movement trajectories, the fit-criteria assessment plots (see Additional file 4) indicated that the linear three-group model was most appropriate for males (BIC = 16938, average posterior probability = 0.93, odds of correct classification = 80.69). Figure 1 shows the general movement trajectories. Profiles included “Highly actives”, “Inactive-sitters”, and “Decreasing activity” profiles. All groups decreased their sleep and increased their sedentary behavior from age 10 to 14. The “Highly actives” were characterized by a combination of relatively high light physical activity and low sedentary behavior compared to other profiles, and increasing time spent in moderate-to-vigorous physical activity. The “Inactive-sitters” had fairly low light physical activity, decreased moderate-to-vigorous physical activity, and had high sedentary behavior. The “Decreasing activity” profile had the least time spent in light physical activity and started with comparable moderate-to-vigorous physical activity as the “Highly actives” but decreased this movement behavior.

The linear five-group model was most appropriate for females (BIC = 13067, average posterior probability = .87, odds of correct classification = 376.23). Profiles included “Highly actives”, “Inactive-sitters”, “Decreasing activity”, “High sleepers”, and “Lightly actives”. The “Highly actives” maintained relatively high moderate-vigorous physical activity compared to other profiles. The “Inactive-sitters” profile was a combination of relatively low moderate-vigorous and high time spent in sedentary behavior compared to the other profiles. The “Decreasing activity” profile had relatively high moderate-vigorous physical activity at age 10 but decreased this movement behavior. The “High sleepers” profile had the most sleep at age 14 compared to all other profiles. The “Lightly actives” profile spent the most time in light physical activity compared to all other profiles, was the only group to increase this movement behavior, and spent a relatively low amount of time in sedentary behavior.

### Domain-specific movement trajectories

**Fig. 2:**
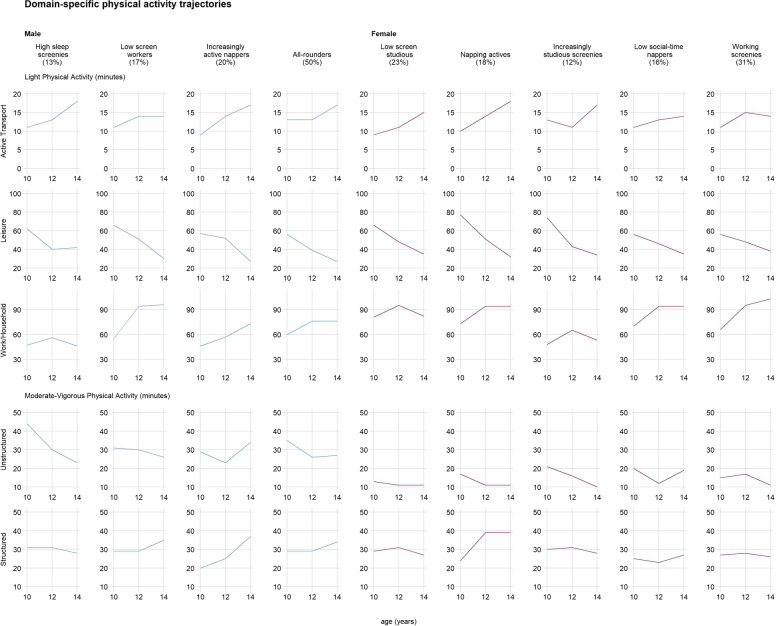
Domain-specific physical activity trajectories

**Fig. 3:**
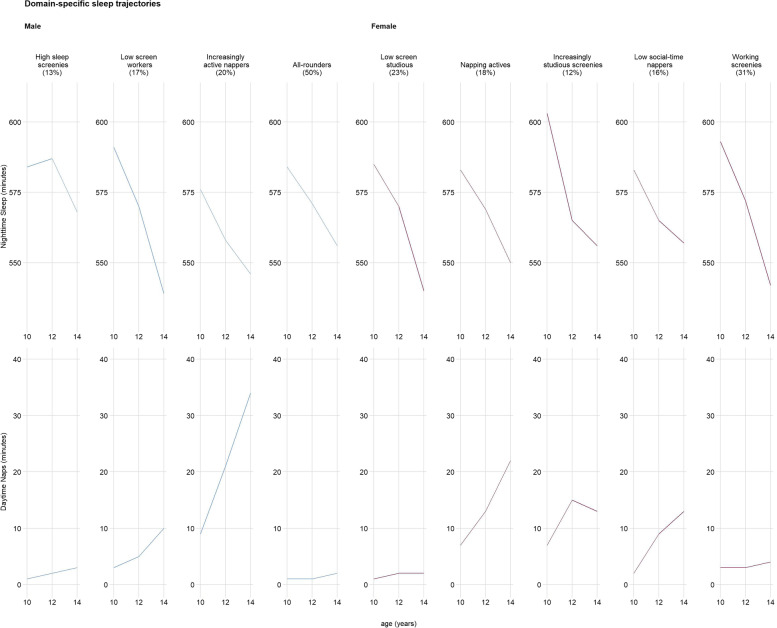
Domain-specific sleep trajectories

**Fig. 4:**
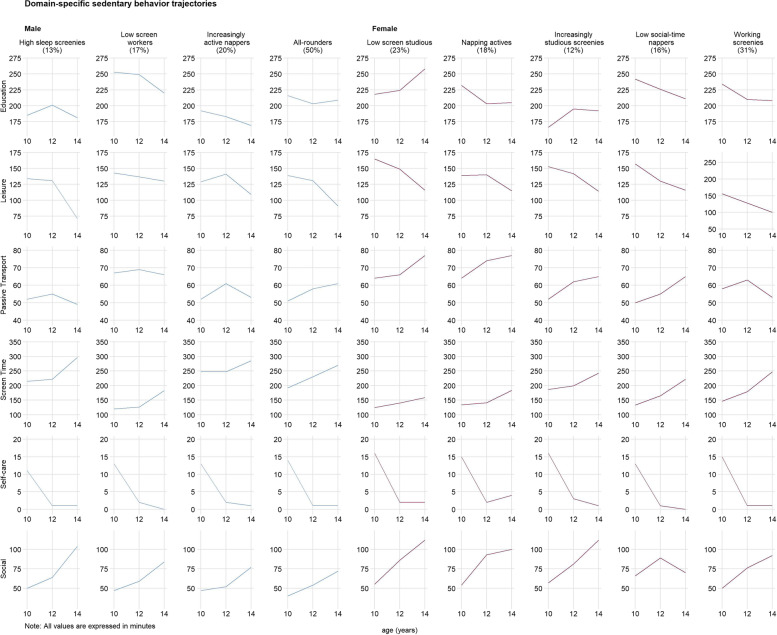
Domain-specific sedentary behavior trajectories

For the domain-specific movement trajectories, the fit-criteria assessment plots indicated a linear four-group model was most appropriate for males (BIC = 69540, average posterior probability = .82, odds of correct classification = 21.07) and a linear five-group model for females (BIC = 67590, average posterior probability = .72, odds of correct classification = 30.05). Plots for these models can be found in Figs. 2, 3 and 4 (see Table 3 for profile characteristics).

**Table 3:** Comparison of domain-specific movement trajectory profile characteristics by sex

Profile Group	Characteristics
**Males**	
High sleep screenies	• Least amount of time in work and household light physical activity compared to other profiles • Steepest decrease in structured moderate-to-vigorous physical activity • Most time in nighttime sleep compared to other profiles • Some napping • Relatively low time in education-based sedentary behavior • Least amount of time in passive transport compared to other profiles • Maintained relatively high time spent in recreational screen activities
Low screen workers	• Relatively most time spent in work and household light physical activity • Slight decrease in unstructured but slight increase in structured moderate-to-vigorous intensity physical activity • Steepest decrease in nighttime sleep • Some napping • Most time spent in education-based sedentary behavior and passive transport compared to other profiles • Least time spent in screen time compared to other profiles
Increasingly active nappers	• Increased structured and unstructured moderate-to-vigorous physical activity • Most time spent napping compared to all other profiles • Least time spent in education-based sedentary behavior compared to all other groups • High amount of recreational screen activities
All-rounders	• Slight decrease in unstructured but slight increase in structured moderate-to-vigorous intensity physical activity • Minimal napping • Moderate amount of work and household light physical activity, nighttime sleep, education-based sedentary behavior, passive transport, and recreational screen activities compared to all other profiles
**Females**	
Low screen studious	• Low unstructured moderate-vigorous physical activity • Minimal naps • Increased education-based sedentary behavior (most time spent compared to all other profiles) • Least time spent in recreational screen activities compared to all other profiles • Relatively high passive transport
Napping actives	• Low unstructured moderate-to-vigorous physical activity but increase in structured moderate-to-vigorous physical activity (most time compared to all other profiles) • Most time spent napping compared to all other profiles • Relatively high passive transport • Relatively low recreational screen activities
Increasingly studious screenies	• Some napping • Increased education-based sedentary behavior (spent the least time compared to all other profiles) • Relatively high recreational screen activities
Low social-time nappers	• Fairly stable structured and unstructured moderate-to-vigorous physical activity • Increased napping • Only group with stable social-based sedentary behavior (least amount of time compared to all other groups)
Working screenies	• Most time in work and household physical activity compared to all other profiles • Limited napping • Only group to decrease passive transport • Relatively high recreational screen activities

### Socioeconomic position as a predictor of movement trajectory profile membership.

For general movement trajectories, socioeconomic position did not predict profile membership for males (see Table 4). Male participants from lower socioeconomic positions were less likely to be in the “All-rounders” profile. Males from lower socioeconomic positions were more likely to engage in a combination of increasing their unstructured and structured moderate-to-vigorous physical activity, napping, and recreational screen activities while spending less time in education-based sedentary behavior compared to their higher socioeconomic peers.

**Table 4:**
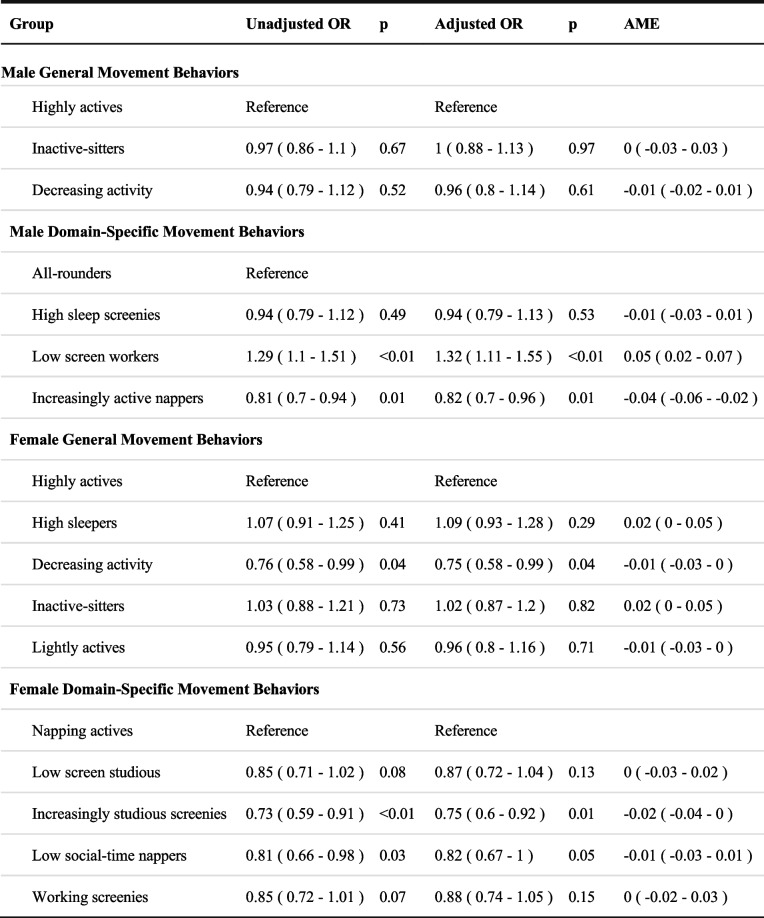
Odds ratios of socioeconomic position predicting movement trajectory membership

Values are expressed as odds ratios and 95% confidence intervals. Trajectories were adjusted for remoteness and Indigenous status

*AME* Average Marginal Effects

Females from lower socioeconomic positions tended to be in the “Decreasing activity” general movement trajectory profile. However, socioeconomic position predicted more memberships for domain-specific movement trajectory profiles. Participants from lower socioeconomic positions were more likely to be in the “Increasingly studious screenies” and “Low social-time nappers” profiles than the “Napping actives” profile. Youth from the “Increasingly studious screenies” and “Low social-time nappers” tended to spend a combination of less time in structured moderate-to-vigorous physical activity, napping, and passive transport but more time in recreational screen activities than those in the “Napping actives” profile.

## Discussion

Our study describes combined movement trajectories in Australian youth from 10 to 14 years old and shows that socioeconomic position was associated with movement trajectories. For general movement trajectory profiles, we found no differences in how males from different socioeconomic positions spend their time but girls from a lower socioeconomic position tend to be a combination of less active and more sedentary than their higher socioeconomic peers. A domain-specific analysis shows the likelihood of how youth from different socioeconomic positions may execute their physical activity, sedentary behavior, and sleep. In line with previous research, all general movement trajectory profiles showed children decreased both their sleep and increased their sedentary behavior as they grew older [54, 55].

We also found that socioeconomic position predicted domain-specific movement trajectory profile membership for Australian children. Males from lower socioeconomic positions tended to spend a combination of more time in activities such as structured and unstructured moderate-to-vigorous physical activity and recreational screen activities rather than the education-based sedentary behavior observed in their higher socioeconomic peers. Females from lower socioeconomic positions tended to displace time in behaviors that have health and well-being benefits, such as moderate-to-vigorous physical activity and napping [25, 56–58], such as moderate-to-vigorous physical activity and napping, with recreational screen time. These differences in domain-specific behaviors align with research on individual movement behaviors that found youth from higher socioeconomic positions spend more time in passive transportation, education-related activities, and structured physical activity [34, 59] while those from lower socioeconomic positions spend more time in recreational screen time [59]. However, contrary to previous research, there was little difference in active transport, unstructured moderate-to-vigorous physical activity and leisure-time (non-screen-based) sedentary behavior for females, structured and unstructured moderate-to-vigorous physical activity in males [31, 32, 35, 60]. These findings highlight the importance of studying movement behaviors in combination because although a child may participate in one healthy or unhealthy movement behavior does not necessarily mean they participate in other healthy or unhealthy movement behaviors across a whole 24-h day.

Future research can investigate if domain-specific movement trajectory profiles are associated with different outcomes in youth 10–14 and identify strategies to address improving these trajectory profiles while considering the needs of youth from different socioeconomic positions. It is important to understand movement trajectories in this age group because they are experiencing many changes such as moving from primary to secondary school and going through puberty [15]. These challenges are often marked by changes in mental and socio-emotional health [13, 61, 62]. Therefore, exploring domain-specific movement trajectories is important because they may influence mental and socio-emotional outcomes differently (e.g., decreasing recreational screen time while increasing structured moderate-vigorous physical activity) [63]. Future studies could identify potential times of day when movement behavior interventions may be useful. For example, previous longitudinal studies have shown that youth from primary to secondary school tend to decrease their moderate-vigorous physical activity while increasing their sedentary behavior during recess, lunchtime and after-school periods [64, 65]. Particularly understanding how youth from various socioeconomic positions spend their time may guide us to develop more specific and targeted interventions that better consider the needs of different subgroups of youth. Addressing domain-specific movement trajectory membership while considering the needs of those from different socioeconomic positions could potentially alleviate some socioeconomic disparities Australian youth face (e.g., differences in academic performance, physical health, and socio-emotional problems) and is a relationship that should be explored [66–70]. Finally, replicating this study’s approach in low-, middle-, and high-income countries is needed to further the existing knowledge base on youth’s movement trajectories.

Although this study provided new insight into how engagement in different movement behaviors change and found that socioeconomic position may predict movement trajectory profiles, some limitations should be considered when interpreting the findings. First, the time-use diaries did not instruct participants to record physical education classes as part of their time-use diaries. Therefore, physical activity may have been underreported across all profiles. There is also no data on the participants' body position (e.g., sitting, standing) in the time-use diary. This may have caused some activities to be coded incorrectly (e.g., a participant reporting “watching television” while standing would have been coded “sedentary” instead of “light physical activity”). Next, the time-use data only captured one day. Consequently, this may not give the most accurate representation of the participant’s week. However, the time-use diaries provided detailed information about the participants’ day [42]. and have been recommended as a tool to investigate health behaviors over time [6, 71, 72]. They have also been shown to be both valid and reliable when collected in large, representative samples [6, 71, 72]. Next, the sample size for weekend participants was insufficient to run a group-based multi-trajectory analysis. Therefore, our results are only generalizable for weekdays. Finally, since the population is representative of Australian youth, our results are not generalizable for low- or middle-income countries.

Despite these limitations, our study had several strengths. Our study used group-based multi-trajectory analysis to identify profiles of youth based on their general movement behaviors and domain-specific movement behaviors which allowed us to use continuous data which overcame the limitation of dichotomizing or categorizing behaviors based on a specific cut-off point (e.g., “meeting guidelines” or “not meeting guidelines”). Additionally, we gained insight regarding how youth’s movement behaviors, in combination, change from primary school to secondary school age. Further, we were able to provide novel insight into a youth's day based on their domain-specific movement behaviors.

## Conclusion

The socioeconomic position of a youth’s family predicted some general and domain-specific movement trajectory profile memberships. There were limited differences in moderate-to-vigorous physical activity in males and nighttime sleep in both sexes. However, those from lower socioeconomic positions tended to participate in a combination of more recreational screen activities and low education-based sedentary behavior for males and low structured physical activity and education-based sedentary behavior for females, activity patterns which may lead to unfavorable outcomes. Future research, interventions and policies should consider targeting domain-specific movement behaviors in combination. Further, the habits, needs, and resources of youth from different socioeconomic positions should be considered when developing recommendations for different socioeconomic position groups.

## Supplementary Information

**Additional file 1.** Categorization of LSAC activities into general and domain-specific movement behaviors.

**Additional file 2.** Describes cut-offs and rationales for outliers.

**Additional file 3.** R code of gbmt analysis.

**Additional file 4.** Fit-criteria assessment plots for choosing general and domain-specific models.

## Data Availability

The data that support the findings of this study are available by application via the DSS Longitudinal Studies Dataverse: http://dx.doi.org/10.26193/BAA3N6. Restrictions apply to the availability of these data, which were used under license for the current study, and so are not publicly available.

## Abbreviation

LSAC: Longitudinal Study of Australian Children
