# Supplementary material for: Socioeconomic position as a predictor of youth's movement trajectory profiles between ages 10 and 14 years
Source: Int J Behav Nutr Phys Act. 2023 Jul 22;20:88. doi: 10.1186/s12966-023-01491-5 (PMC10363305; doi:10.1186/s12966-023-01491-5)
Supplement: Supplementary file 1 — Additional file 1. Categorization of LSAC activities into general and domain-specific movement behaviors. [file 12966_2023_1491_MOESM1_ESM.pdf]

Additional file 1.

Activity Code	Activity	LSAC category	General Movement Behaviour Category	Domain-Specific Category
454	Window shopping	Active Activities	Light-Physical Activity	Unstructured Leisure-time Physical Activity
463	Attendance at musuem/exhibitio	Active Activities	Light-Physical Activity	Unstructured Leisure-time Physical Activity
464	Attendance at zoo/animal park/l	Active Activities	Light-Physical Activity	Unstructured Leisure-time Physical Activity
465	Attendance at other mass even	Active Activities	Light-Physical Activity	Unstructured Leisure-time Physical Activity
466	Going out nec	Active Activities	Light-Physical Activity	Unstructured Leisure-time Physical Activity
491	Active activities nec	Active Activities	Light-Physical Activity	Unstructured Leisure-time Physical Activity
450	Shopping	Active Activities	Light-Physical Activity	Unstructured Leisure-time Physical Activity
451	Shopping	Active Activities	Light-Physical Activity	Unstructured Leisure-time Physical Activity
1	Retailing	Work	Light-Physical Activity	Work/Household Physical Activity
11	Hospitality (including fast food)	Work	Light-Physical Activity	Work/Household Physical Activity
31	Labourers and related workers	Work	Light-Physical Activity	Work/Household Physical Activity
41	Gardening/lawn mowing	Work	Light-Physical Activity	Work/Household Physical Activity
51	Babysitting	Work	Light-Physical Activity	Work/Household Physical Activity
61	Apprenticeships/trades persons	Work	Light-Physical Activity	Work/Household Physical Activity
71	Working in a family business or	Work	Light-Physical Activity	Work/Household Physical Activity
82	Umpring (work)	Work	Light-Physical Activity	Work/Household Physical Activity
83	Car washing (work)	Work	Light-Physical Activity	Work/Household Physical Activity
84	Animal care (work)	Work	Light-Physical Activity	Work/Household Physical Activity
91	Volunteering (work)	Work	Light-Physical Activity	Work/Household Physical Activity
201	Cleaning teeth	Personal Care/Medical/Health Care	Light-Physical Activity	Work/Household Physical Activity
211	Showering/bathing	Personal Care/Medical/Health Care	Light-Physical Activity	Work/Household Physical Activity
221	Getting dressed/getting ready	Personal Care/Medical/Health Care	Light-Physical Activity	Work/Household Physical Activity
231	Personal care nec	Personal Care/Medical/Health Care	Light-Physical Activity	Work/Household Physical Activity
301	Cleaning/tidying	Chores	Light-Physical Activity	Work/Household Physical Activity
311	Laundry/clothes care	Chores	Light-Physical Activity	Work/Household Physical Activity
321	Food/drink preparation	Chores	Light-Physical Activity	Work/Household Physical Activity
331	Food/drink clean up	Chores	Light-Physical Activity	Work/Household Physical Activity
341	Gardenin (maintenance chores)	Chores	Light-Physical Activity	Work/Household Physical Activity
342	Cleaning grounds/garage/shed/	Chores	Light-Physical Activity	Work/Household Physical Activity
344	Pool care (chores	Chores	Light-Physical Activity	Work/Household Physical Activity
351	Animal Care (work)	Chores	Light-Physical Activity	Work/Household Physical Activity
361	Home maintenance	Chores	Light-Physical Activity	Work/Household Physical Activity
362	Design/Home Improvement	Chores	Light-Physical Activity	Work/Household Physical Activity
363	Heat/water/power upkeep	Chores	Light-Physical Activity	Work/Household Physical Activity
364	Car/boat/bike care	Chores	Light-Physical Activity	Work/Household Physical Activity
365	Selling/disposing of household a	Chores	Light-Physical Activity	Work/Household Physical Activity
366	Rubbish/Recyling	Chores	Light-Physical Activity	Work/Household Physical Activity
367	Packing	Chores	Light-Physical Activity	Work/Household Physical Activity
368	Household management Other	Chores	Light-Physical Activity	Work/Household Physical Activity
371	Taking care of siblings (chores)	Chores	Light-Physical Activity	Work/Household Physical Activity
381	Chores nec	Chores	Light-Physical Activity	Work/Household Physical Activity
901	By foot	Travel	Light-Physical Activity	Active Transportation
911	By bike, scooter, skateboard, et	Travel	Light-Physical Activity	Active Transportation
401	Archery/Shooting sports	Active Activities	Moderate-to-Vigorous Physical Activity	Structured Leisure-time Physical Activity
402	Athletics/Gymnastics	Active Activities	Moderate-to-Vigorous Physical Activity	Structured Leisure-time Physical Activity
403	Fitness/Gym/Exercise	Active Activities	Moderate-to-Vigorous Physical Activity	Structured Leisure-time Physical Activity
404	Ball Sports	Active Activities	Moderate-to-Vigorous Physical Activity	Structured Leisure-time Physical Activity
405	Martial arts/Dancing	Active Activities	Moderate-to-Vigorous Physical Activity	Structured Leisure-time Physical Activity
406	Motor Sports/Roller Sports/Cycl	Active Activities	Moderate-to-Vigorous Physical Activity	Structured Leisure-time Physical Activity
407	Water/Ice/Snow Sports	Active Activities	Moderate-to-Vigorous Physical Activity	Structured Leisure-time Physical Activity
408	Organised team sports and trai	Active Activities	Moderate-to-Vigorous Physical Activity	Structured Leisure-time Physical Activity
411	Archery/shooting sports (individ	Active Activities	Moderate-to-Vigorous Physical Activity	Structured Leisure-time Physical Activity
412	Athletics/Gymnastics (individua	Active Activities	Moderate-to-Vigorous Physical Activity	Structured Leisure-time Physical Activity
413	Fitness/Gym/Exercise (individu	Active Activities	Moderate-to-Vigorous Physical Activity	Structured Leisure-time Physical Activity
414	Martial arts/Dancing (individual)	Active Activities	Moderate-to-Vigorous Physical Activity	Structured Leisure-time Physical Activity
415	Motor Sports/Roller Sports/Cycl	Active Activities	Moderate-to-Vigorous Physical Activity	Structured Leisure-time Physical Activity
416	Ball sports (individual)	Active Activities	Moderate-to-Vigorous Physical Activity	Structured Leisure-time Physical Activity
417	Water/Ice/Snow Sports (individu	Active Activities	Moderate-to-Vigorous Physical Activity	Structured Leisure-time Physical Activity
418	Organised individual sports and	Active Activities	Moderate-to-Vigorous Physical Activity	Structured Leisure-time Physical Activity
430	Walking pets/playing with pets	Active Activities	Moderate-to-Vigorous Physical Activity	Structured Leisure-time Physical Activity
440	Active club activities	Active Activities	Moderate-to-Vigorous Physical Activity	Structured Leisure-time Physical Activity
421	Archery/Shooting sports (unstru	Active Activities	Moderate-to-Vigorous Physical Activity	Unstructured Leisure-time Physical Activity
422	Athletics/Gymnastics (unstructu	Active Activities	Moderate-to-Vigorous Physical Activity	Unstructured Leisure-time Physical Activity
423	Fitness/Gym/Exercise (unstruct	Active Activities	Moderate-to-Vigorous Physical Activity	Unstructured Leisure-time Physical Activity
424	Ball sports (unstructured)	Active Activities	Moderate-to-Vigorous Physical Activity	Unstructured Leisure-time Physical Activity
425	Martial arts/Dancing (unstructur	Active Activities	Moderate-to-Vigorous Physical Activity	Unstructured Leisure-time Physical Activity
426	Motor Sports/Roller Sports/Cycl	Active Activities	Moderate-to-Vigorous Physical Activity	Unstructured Leisure-time Physical Activity
427	Water/Ice/Snow Sports (unstruc	Active Activities	Moderate-to-Vigorous Physical Activity	Unstructured Leisure-time Physical Activity
428	Unstructured active play Other	Active Activities	Moderate-to-Vigorous Physical Activity	Unstructured Leisure-time Physical Activity
21	Clerical/office	Work	Sedentary Behaviour	Education-Based Sedentary Behaviour
81	Work Other	Work	Sedentary Behaviour	Education-Based Sedentary Behaviour
501	Private music lessons/practice,	Non-Active Activities	Sedentary Behaviour	Education-Based Sedentary Behaviour
521	Playing musical instruments or s	Non-Active Activities	Sedentary Behaviour	Education-Based Sedentary Behaviour
531	Reading or being red to for leisu	Non-Active Activities	Sedentary Behaviour	Education-Based Sedentary Behaviour
541	Chess, cards , paper, and board	Non-Active Activities	Sedentary Behaviour	Education-Based Sedentary Behaviour
581	Doing homework (not via electro	Non-Active Activities	Sedentary Behaviour	Education-Based Sedentary Behaviour
601	Doing homework (electronic dev	Electronic Device Use	Sedentary Behaviour	Education-Based Sedentary Behaviour
701	School lessons	School Lessons	Sedentary Behaviour	Education-Based Sedentary Behaviour
281	Person care/Medical/Health car	Personal Care/Medical/Health Care	Sedentary Behaviour	Leisure-time Sedentary Behaviour
312	Clothes making	Chores	Sedentary Behaviour	Leisure-time Sedentary Behaviour
981	Filling out the diary	Non-Active Activities	Sedentary Behaviour	Leisure-time Sedentary Behaviour
481	Attending live sporting events	Active Activities	Sedentary Behaviour	Leisure-time Sedentary Behaviour
462	Attendance at concert/theatre	Active Activities	Sedentary Behaviour	Leisure-time Sedentary Behaviour
101	Eating/drinking	Eating/drinking	Sedentary Behaviour	Leisure-time Sedentary Behaviour
511	Listening to music	Non-Active Activities	Sedentary Behaviour	Leisure-time Sedentary Behaviour
542	Games of chance/gambling	Non-Active Activities	Sedentary Behaviour	Leisure-time Sedentary Behaviour
543	Hobbies, collections	Non-Active Activities	Sedentary Behaviour	Leisure-time Sedentary Behaviour
544	Handwork crafts (excl. clothes r	Non-Active Activities	Sedentary Behaviour	Leisure-time Sedentary Behaviour

545	Arts	Non-Active Activities	Sedentary Behaviour	Leisure-time Sedentary Behaviour
548	Unstructure non-active play nec	Non-Active Activities	Sedentary Behaviour	Leisure-time Sedentary Behaviour
551	Attend courses (exlcuding scho	Non-Active Activities	Sedentary Behaviour	Leisure-time Sedentary Behaviour
552	Clubs	Non-Active Activities	Sedentary Behaviour	Leisure-time Sedentary Behaviour
555	Religious groups	Non-Active Activities	Sedentary Behaviour	Leisure-time Sedentary Behaviour
561	Doing nothing	Non-Active Activities	Sedentary Behaviour	Leisure-time Sedentary Behaviour
591	Non-active activites nec	Non-Active Activities	Sedentary Behaviour	Leisure-time Sedentary Behaviour
452	Purchasing consumer goods	Active Activities	Sedentary Behaviour	Leisure-time Sedentary Behaviour
453	Purchasing durable goods	Active Activities	Sedentary Behaviour	Leisure-time Sedentary Behaviour
455	Purchasing repair services	Active Activities	Sedentary Behaviour	Leisure-time Sedentary Behaviour
456	Purchasing administrative servi	Active Activities	Sedentary Behaviour	Leisure-time Sedentary Behaviour
457	Purchasing personal care servi	Active Activities	Sedentary Behaviour	Leisure-time Sedentary Behaviour
458	Purchasing other services	Active Activities	Sedentary Behaviour	Leisure-time Sedentary Behaviour
471	Religious practice	Active Activities	Sedentary Behaviour	Leisure-time Sedentary Behaviour
472	Weddings, funeral, rites of pass	Active Activities	Sedentary Behaviour	Leisure-time Sedentary Behaviour
473	Religious activites/ ritual cerem	Active Activities	Sedentary Behaviour	Leisure-time Sedentary Behaviour
921	By private motor vehicle/bike	Travel	Sedentary Behaviour	Passive Transportation
931	By public/chartered transport	Travel	Sedentary Behaviour	Passive Transportation
941	Travel nec	Travel	Sedentary Behaviour	Passive Transportation
461	Attendance at movies/cinema	Active Activities	Sedentary Behaviour	Recreational Screen Time
611	Playing games (electornic devic	Electronic Device Use	Sedentary Behaviour	Recreational Screen Time
612	Playing games (electornic devic	Electronic Device Use	Sedentary Behaviour	Recreational Screen Time
621	Watching TV programs or movi	Electronic Device Use	Sedentary Behaviour	Recreational Screen Time
631	Spending time on social networ	Electronic Device Use	Sedentary Behaviour	Recreational Screen Time
641	Downloading/posting media	Electronic Device Use	Sedentary Behaviour	Recreational Screen Time
651	Internet shopping	Electronic Device Use	Sedentary Behaviour	Recreational Screen Time
661	General Internet browsing	Electronic Device Use	Sedentary Behaviour	Recreational Screen Time
671	Creating/maintaining websites	Electronic Device Use	Sedentary Behaviour	Recreational Screen Time
681	General application use	Electronic Device Use	Sedentary Behaviour	Recreational Screen Time
691	Electornic devisue use nec	Electronic Device Use	Sedentary Behaviour	Recreational Screen Time
841	Texting/emailing	Communication	Sedentary Behaviour	Recreational Screen Time
851	Online chatting/Instant messagi	Communication	Sedentary Behaviour	Recreational Screen Time
241	Doctor	Personal Care/Medical/Health Care	Sedentary Behaviour	Self Care
251	Dentist/Orthodontist	Personal Care/Medical/Health Care	Sedentary Behaviour	Self Care
261	Physiotherapist/Chiropractor	Personal Care/Medical/Health Care	Sedentary Behaviour	Self Care
271	Medical/Health care	Personal Care/Medical/Health Care	Sedentary Behaviour	Self Care
802	Talking face-to-face	Communication	Sedentary Behaviour	Social-Based Sedentary Behaviour
811	Talking on a landline phone	Communication	Sedentary Behaviour	Social-Based Sedentary Behaviour
821	Talking on a mobile phone	Communication	Sedentary Behaviour	Social-Based Sedentary Behaviour
831	Video chatting	Communication	Sedentary Behaviour	Social-Based Sedentary Behaviour
861	Non-verbal interaction	Communication	Sedentary Behaviour	Social-Based Sedentary Behaviour
871	Negative face-to-face commica	Communication	Sedentary Behaviour	Social-Based Sedentary Behaviour
881	Communication nec	Communication	Sedentary Behaviour	Social-Based Sedentary Behaviour
571	Sleeping/napping (not end of da	Non-Active Activities	Sleep	Daytime Naps
971	Illegal activities	Other	Uncodeable	Uncodeable
991	Other	Other	Uncodeable	Uncodeable
998	Uncodeable activity	Uncodeable Activity	Uncodeable	Uncodeable
