# Supplementary material for: Socioeconomic position as a predictor of youth's movement trajectory profiles between ages 10 and 14 years
Source: Int J Behav Nutr Phys Act. 2023 Jul 22;20:88. doi: 10.1186/s12966-023-01491-5 (PMC10363305; doi:10.1186/s12966-023-01491-5)
Supplement: Supplementary file 2 — Additional file 2. Describes cut-offs and rationales for outliers. [file 12966_2023_1491_MOESM2_ESM.pdf]

Additional file 2. Outlier decisions.

**Domain-Specific Movement Behavior**	**Cut-off Time for Outliers**	**Rationale**	**Updated Included Participants**
Active Transportation (Age 10)	>= 300 min	Doesn't seem like a reasonable time for a 10-year old to be walking and timing doesn't make it look like the family went for a bushwalk (e.g., one participant walked from 19:30-1:00)	Original: 3,309<br>Excluded: 3<br>Updated: 3,306
Daytime Naps (Age 10)	OK	*Going to change "outlier naps' >240 min to nighttime sleep as these naps started before 5am with participants waking up for toilet/drink breaks and then "napping" again	
Education-Based Sedentary Behavior (Age 10)	OK		
Leisure-Time Sedentary Behavior (Age 10)	>550 min	Participants had abnormally long eating times (4-8+ hours) or many activities were a secondary activity to "not in coder"	Excluded: 11<br>Updated: 3,295
Passive Transportation (Age 10)	>= 302 min	Participants were on the road all day, mainly broken up my eating breaks. This suggests the family was going on a long trip which would not	Excluded: 32<br>Updated: 3,263

		be a typical day	
Recreational Screen Time (Age 10)	OK	There are some very high numbers, but it's reasonable to think that some 10-year olds spend their weekends watching TV and playing games all day	
Self-Care Sedentary Behavior (Age 10)	>= 90 min	These participants either reported being sick, having a doctor's appointment, or visiting the hospital; exclude since this is not indicative of a typical day	Excluded: 80<br>Updated: 3,183
Social-Based SB (Age 10)	OK		
Structured MVPA (Age 10)	>=350	Participants appeared to be at competitions or camps (e.g., MVPA for 2 hours, eating for 30 minutes - pattern repeated throughout the whole day)<br>Even if this is a typical weekend, it is reasonable to think that the participants were active the entire time (e.g. resting between event competitions, waiting for their turn to compete, sitting on the bench, etc.)	Excluded: 7<br>Updated: 3,176
Unstructured LPA (Age 10)	> 660 - however, this participant was already eliminated by	Abnormal day; looks like diary wasn't filled out properly	Excluded: 0<br>Updated: 3,176

	one of the above criteria		
Unstructured MVPA (Age 10)	> = 350	The day looked like it could have been normal, but very long bouts were sometimes paired with SB. It is reasonable to assume that these participants were not participating in MVPA the entire time.<br>This cut-off number was chosen in line with structured MVPA	Excluded: 3<br>Updated: 3,173
Work/Household LPA (Age 10)	>= 800	One participant only recorded 1 activity for the entire day, this is extremely unlikely to be a true representation of the day. All other participants with high volumes of work/household LPA were doing chores all day, but their days were split up with appropriate breaks. Therefore, they were not excluded.	Excluded: 1<br>Updated: 3,172
Nighttime Sleep (Age 10)	>= 1000<br>&<br><200	1 participant met this criteria. They slept over 100 min longer than the closest cluster of high duration sleepers.<br>A couple participants with very low sleep, appears that TUD was incorrectly filled	Excluded: 3 (1 long, 2 short)<br>Updated: 3,169

Active Transportation (Age 12)	>= 300 min	Same as age 10	Original: 3,024<br>Excluded: 4<br>Updated: 3,020
Daytime Naps (Age 12)	OK	Same as age 10	
Education (Age 12)	OK	Same as age 10	
Leisure Time SB (Age 12)	OK	Nothing abnormal	
Passive Transportation (Age 12)	>330 min	Same as above	Excluded: 21<br>Updated: 2,991
Recreational Screen Time (Age 12)	>1000	Abnormal circumstance for not going to school reported	Excluded: 1<br>Updated: 2,990
Self care (Age 12)	>= 90 min	Same as above	Excluded: 20<br>Updated: 2,970
Social (Age 12)	>800 min	Same activity repeated in succession without other activities	Excluded: 1<br>Updated: 2,969
Structured MVPA (Age 12)	>=350	Same as above	Excluded: 6<br>Updated: 2,963
Unstructured LPA (Age 12)	OK	High volume of time, but reasonable activities done throughout the day	
Unstructured MVPA (Age 12)	>=350	Same as above	Excluded: 10<br>Updated: 2,953
Work and Household LPA (Age 12)	>=800	Diary entry appears incorrect	Excluded: 1<br>Updated: 2,952
Nighttime Sleep (Age 12)	<200	Very low sleep and days looked atypical	Excluded: 2<br>Updated: 2,950
Active Transportation (Age 14)	>=300 min	Abnormal walking periods not broken up	Original: 2,736<br>Excluded: 2

		by anything/sometimes paired with sedentary secondary activities or uncodeable main activities	Updated: 2,734
Daytime Naps (Age 14)	>700 min	Abnormal diaries and patterns	Excluded: 1<br>Updated:2,733
Education (Age 14)	OK		
Leisure SB (Age 14)	>700 min	Diary incomplete	Excluded: 1<br>Updated: 2,732
Passive Transportation (Age 14)	>340 min		Excluded:25<br>Updated: 2,707
Recreational Screen Time (Age 14)	>1000	All participants had school, but did not attend	Excluded: 3<br>Updated: 2,704
Self-Care (Age 14)	>=90 min	Same as above	Excluded: 36<br>Updated: 2,670
Social (Age 14)	>750 min	Same as above	Excluded: 3<br>Updated: 2,667
Structured MVPA (Age 14)	>=350 min	Same as above	Excluded: 9<br>Updated: 2,658
Unstr. LPA (Age 14)	OK		
Unstr. MVPA (Age 14)	>=350 min	Same as above	Excluded: 3<br>Updated: 2,655
Work/Household LPA (Age 14)	Looks good		
Nighttime Sleep (Age 14)	>1000<br>&<br><100	very long and participant did not go to school on a school day;<br>20 minute sleep time -TUD looked incorrect and it is reasonable to think	Excluded: 2 (1 meeting each criteria)<br>Updated: 2,653

		this is not a typical day	

Note: active transportation values at all ages were from total number of valid time-use diaries used at each timepoint
