# Supplementary material for: Socioeconomic position as a predictor of youth's movement trajectory profiles between ages 10 and 14 years
Source: Int J Behav Nutr Phys Act. 2023 Jul 22;20:88. doi: 10.1186/s12966-023-01491-5 (PMC10363305; doi:10.1186/s12966-023-01491-5)
Supplement: Supplementary file 3 — Additional file 3. R code of gbmt analysis. [file 12966_2023_1491_MOESM3_ESM.pdf]

Additional file 3. gbmt code

```
#install.packages("foreach")
#install.packages("doParallel")
install.packages("haven")
install.packages("remotes")
remotes::install_github("alessandromagrini/gbmt")
install.packages("tidyverse")
install.packages("foreach")
install.packages("doParallel")
install.packages("data.table")
install.packages("reshape2")
install.packages("ggplot2")
install.packages("fmsb")
install.packages("dplyr")
install.packages("devtools")
library(gbmt)
library(tidyverse)
library(foreach)
library(doParallel)
library(data.table)
library(reshape2)
library(ggplot2)
library(fmsb)
library(dplyr)
library(devtools)
library(haven)


# Read the No Outliers Dataset
df <-
  haven::read_dta(
    "Z:\\LSAC
dataset\\Study_2\\Study_2\\No_outliers_Multi_Trajectory_Analysis_Domain_Specific_Movement
_Behaviours.dta"
  )

#GENERAL WEEKDAYS DATAFRAME
df_gen_weekday <- df[df$day_of_week_at_10 == 1,]
save(df_gen_weekday, file = 'df_gen_weekday.Rdata')

#GENERAL WEEKDAYS MALE GBMT
df_males_gen_weekday <- df_gen_weekday[df_gen_weekday$Sex ==1,]
save(df_males_gen_weekday, file = 'df_males_gen_weekday.Rdata')
```

```
df_long <- df_males_gen_weekday %>%
  distinct(hicid, .keep_all = TRUE) %>%
  pivot_longer(
    cols = ends_with(c("10", "12", "14")),
    names_to = c(".value", "time"),
    names_pattern = "(.*?)_(\\d\\d)"
  ) %>%
  mutate(across(where(is.numeric), ~ if_else(.x == 0, 0.01, .x)),
         time = as.integer(time)) %>%
  as.data.frame()

variables = c("LPA_at", "MVPA_at", "SB_at", "sleep_at")

df_long_reduced <- df_long %>%
  select(variables, time, hicid)

# Setup the backend cluster
cl <- makeCluster(detectCores() - 1) # you can reduce this if needed
registerDoParallel(cl)

# Define the function to process
get_metrics <- function(df, variables) {
  foreach(ng = rep(2:6, 2), d = c(rep(1, 5), rep(2, 5))) %dopar% {
    tryCatch({
      set.seed(42)
      res <-
        gbmt::gbmt(
          x.names = variables,
          unit = "hicid",
          time = "time",
          d = d,
          ng = ng,
          data = df,
          nstart = 10,
          pruning = FALSE
        )
      res
    },
    error =  function(e)
      print(paste('e:', e)))
  }
}
```

```
# Run the function
results_male_gen_weekday <- get_metrics(df_long_reduced, variables)

# Stop the backend cluster
stopCluster(cl)

# Save results
save(results_male_gen_weekday, file = 'results_male_gen_weekday.Rdata')

do.call(rbind, lapply(results_male_gen_weekday, function(x) {
  y <- x$ic
  y["ng"] <- x$call$ng
  y["d"] <- x$call$d
  y
}))

# Make Universal CSV files for models 1-5 for FCAP analysis
make_csvs <- function(i) {
  grps <- results_male_gen_weekday[[i]]$call$ng
  grpcols <- grps + 2
  first_line <-
    paste(
      results_male_gen_weekday[[i]]$ic[["aic"]],
      results_male_gen_weekday[[i]]$ic[["bic"]],
      paste0(results_male_gen_weekday[[i]]$logLik, "\n"),
      sep = ","
    )
  out_table <-
    merge(results_male_gen_weekday[[i]]$posterior,
          results_male_gen_weekday[[i]]$assign,
          by = "row.names")[, 2:grpcols]
  filename <-
    paste0("./FCAP/FCAP_male_gen_weekday/FCAP CSV Files_models1_5/",
           "universal_",
           grps,
           ".csv")
  cat(first_line, file = filename)
  write.table(
    out_table,
    file = filename,
    append = TRUE,
    sep = ",",
    row.names = FALSE,
    col.names = FALSE
```

```
  )
}

for (n in 1:5) {
  try{
    make_csvs(n)
  }
}

# Make Universal CSV files for models 6-10 for FCAP analysis
make_csvs <- function(i) {
  grps <- results_male_gen_weekday[[i]]$call$ng
  grpcols <- grps + 2
  first_line <-
    paste(
      results_male_gen_weekday[[i]]$ic[["aic"]],
      results_male_gen_weekday[[i]]$ic[["bic"]],
      paste0(results_male_gen_weekday[[i]]$logLik, "\n"),
      sep = ","
    )
  out_table <-
    merge(results_male_gen_weekday[[i]]$posterior,
          results_male_gen_weekday[[i]]$assign,
          by = "row.names")[, 2:grpcols]
  filename <-
    paste0("./FCAP/FCAP_male_gen_weekday/FCAP CSV Files_models6_10/",
           "universal_",
           grps,
           ".csv")
  cat(first_line, file = filename)
  write.table(
    out_table,
    file = filename,
    append = TRUE,
    sep = ",",
    row.names = FALSE,
    col.names = FALSE
  )
}

for (n in 6:10) {
  try{
    make_csvs(n)
  }
```

```
}

#GENERAL WEEKDAYS female GBMT
df_females_gen_weekday <- df_gen_weekday[df_gen_weekday$Sex ==2,]
save(df_females_gen_weekday, file = 'df_females_gen_weekday.Rdata')

df_long <- df_females_gen_weekday %>%
  distinct(hicid, .keep_all = TRUE) %>%
  pivot_longer(
    cols = ends_with(c("10", "12", "14")),
    names_to = c(".value", "time"),
    names_pattern = "(.*?)_(\\d\\d)"
  ) %>%
  mutate(across(where(is.numeric), ~ if_else(.x == 0, 0.01, .x)),
         time = as.integer(time)) %>%
  as.data.frame()

variables = c("LPA_at", "MVPA_at", "SB_at", "sleep_at")

df_long_reduced <- df_long %>%
  select(variables, time, hicid)

# Setup the backend cluster
cl <- makeCluster(detectCores() - 1) # you can reduce this if needed
registerDoParallel(cl)

# Define the function to process
get_metrics <- function(df, variables) {
  foreach(ng = rep(2:6, 2), d = c(rep(1, 5), rep(2, 5))) %dopar% {
    tryCatch({
      set.seed(42)
      res <-
        gbmt::gbmt(
          x.names = variables,
          unit = "hicid",
          time = "time",
          d = d,
          ng = ng,
          data = df,
          nstart = 10,
          pruning = FALSE
        )
      res
```

```
    },
    error =  function(e)
      print(paste('e:', e)))
  }
}

# Run the function
results_female_gen_weekday <- get_metrics(df_long_reduced, variables)

# Stop the backend cluster
stopCluster(cl)

# Save results
save(results_female_gen_weekday, file = 'results_female_gen_weekday.Rdata')

do.call(rbind, lapply(results_female_gen_weekday, function(x) {
  y <- x$ic
  y["ng"] <- x$call$ng
  y["d"] <- x$call$d
  y
}))

# Make Universal CSV files for models 1-5 for FCAP analysis
make_csvs <- function(i) {
  grps <- results_female_gen_weekday[[i]]$call$ng
  grpcols <- grps + 2
  first_line <-
    paste(
      results_female_gen_weekday[[i]]$ic[["aic"]],
      results_female_gen_weekday[[i]]$ic[["bic"]],
      paste0(results_female_gen_weekday[[i]]$logLik, "\n"),
      sep = ","
    )
  out_table <-
    merge(results_female_gen_weekday[[i]]$posterior,
          results_female_gen_weekday[[i]]$assign,
          by = "row.names")[, 2:grpcols]
  filename <-
    paste0("./FCAP/FCAP_female_gen_weekday/FCAP CSV Files_models1_5/",
           "universal_",
           grps,
           ".csv")
  cat(first_line, file = filename)
  write.table(
```

```
    out_table,
    file = filename,
    append = TRUE,
    sep = ",",
    row.names = FALSE,
    col.names = FALSE
  )
}

for (n in 1:5) {
  try{
    make_csvs(n)
  }
}

# Make Universal CSV files for models 6-10 for FCAP analysis
make_csvs <- function(i) {
  grps <- results_female_gen_weekday[[i]]$call$ng
  grpcols <- grps + 2
  first_line <-
    paste(
      results_female_gen_weekday[[i]]$ic[["aic"]],
      results_female_gen_weekday[[i]]$ic[["bic"]],
      paste0(results_female_gen_weekday[[i]]$logLik, "\n"),
      sep = ","
    )
  out_table <-
    merge(results_female_gen_weekday[[i]]$posterior,
          results_female_gen_weekday[[i]]$assign,
          by = "row.names")[, 2:grpcols]
  filename <-
    paste0("./FCAP/FCAP_female_gen_weekday/FCAP CSV Files_models6_10/",
           "universal_",
           grps,
           ".csv")
  cat(first_line, file = filename)
  write.table(
    out_table,
    file = filename,
    append = TRUE,
    sep = ",",
    row.names = FALSE,
    col.names = FALSE
  )
```

```
}

for (n in 6:10) {
  try{
    make_csvs(n)
  }


# DOMAIN-SPECIFIC WEEKDAYS
df_domsp_weekday <- df[df$day_of_week_at_10 == 1,]
save(df_domsp_weekday, file = 'df_domsp_weekday.Rdata')

#DOMAIN-SPECIFIC WEEKDAYS MALES
df_male_domsp_weekday <- df_domsp_weekday[df_domsp_weekday$Sex == 1,]
save(df_male_domsp_weekday, file = 'df_male_domsp_weekday.Rdata')

df_long <- df_male_domsp_weekday %>%
  distinct(hicid, .keep_all = TRUE) %>%
  pivot_longer(
    cols = ends_with(c("10", "12", "14")),
    names_to = c(".value", "time"),
    names_pattern = "(.*?)_(\\d\\d)"
  ) %>%
  mutate(across(where(is.numeric), ~ if_else(.x == 0, 0.01, .x)),
         time = as.integer(time)) %>%
  as.data.frame()

variables = c(
  "active_transport_at",
  "daytime_naps_at",
  "education_SB_at",
  "leisure_time_SB_at",
  "passive_transport_at",
  "screen_time_at",
  "self_care_SB_at",
  "social_based_SB_at",
  "structured_MVPA_at",
  "unstructured_LPA_at",
  "unstructured_MVPA_at",
  "household_LPA_at",
  "nighttime_sleep_at"
)

df_long_reduced <- df_long %>%
```

```
  select(variables, time, hicid)

# Setup the backend cluster
cl <- makeCluster(detectCores() - 1) # you can reduce this if needed
registerDoParallel(cl)

# Define the function to process
get_metrics <- function(df, variables) {
  foreach(ng = rep(2:6, 2), d = c(rep(1, 5), rep(2, 5))) %dopar% {
    tryCatch({
      set.seed(42)
      res <-
        gbmt::gbmt(
          x.names = variables,
          unit = "hicid",
          time = "time",
          d = d,
          ng = ng,
          data = df,
          nstart = 10,
          pruning = FALSE
        )
      res
    },
    error =  function(e)
      0)
  }
}

# Run the function
results_male_domsp_weekday <- get_metrics(df_long_reduced, variables)

# Stop the backend cluster
stopCluster(cl)

# Save results
save(results_male_domsp_weekday, file = 'results_male_domsp_weekday.Rdata')

# Put the results in a table
do.call(rbind, lapply(results_male_domsp_weekday, function(x) {
  y <- x$ic
  y["ng"] <- x$call$ng
  y["d"] <- x$call$d
  y
```

```
}))

# Make Universal CSV files for models 1-5 for FCAP analysis
make_csvs <- function(i) {
  grps <- results_male_domsp_weekday[[i]]$call$ng
  grpcols <- grps + 2
  first_line <-
    paste(
      results_male_domsp_weekday[[i]]$ic[["aic"]],
      results_male_domsp_weekday[[i]]$ic[["bic"]],
      paste0(results_male_domsp_weekday[[i]]$logLik, "\n"),
      sep = ","
    )
  out_table <-
    merge(results_male_domsp_weekday[[i]]$posterior,
          results_male_domsp_weekday[[i]]$assign,
          by = "row.names")[, 2:grpcols]
  filename <-
    paste0(
      "./FCAP/FCAP_male_domsp_weekday/FCAP CSV Files_models1_5/",
      "universal_",
      grps,
      ".csv"
    )
  cat(first_line, file = filename)
  write.table(
    out_table,
    file = filename,
    append = TRUE,
    sep = ",",
    row.names = FALSE,
    col.names = FALSE
  )
}

for (n in 1:5) {
  try{
    make_csvs(n)
  }
}

# Make Universal CSV files for models 6-10 for FCAP analysis
make_csvs <- function(i) {
  grps <- results_male_domsp_weekday[[i]]$call$ng
```

```
  grpcols <- grps + 2
  first_line <-
    paste(
      results_male_domsp_weekday[[i]]$ic[["aic"]],
      results_male_domsp_weekday[[i]]$ic[["bic"]],
      paste0(results_male_domsp_weekday[[i]]$logLik, "\n"),
      sep = ","
    )
  out_table <-
    merge(results_male_domsp_weekday[[i]]$posterior,
          results_male_domsp_weekday[[i]]$assign,
          by = "row.names")[, 2:grpcols]
  filename <-
    paste0(
      "./FCAP/FCAP_male_domsp_weekday/FCAP CSV Files_models6_10/",
      "universal_",
      grps,
      ".csv"
    )
  cat(first_line, file = filename)
  write.table(
    out_table,
    file = filename,
    append = TRUE,
    sep = ",",
    row.names = FALSE,
    col.names = FALSE
  )
}

for (n in 6:10) {
  try{
    make_csvs(n)
  }
}

#DOMAIN-SPECIFIC WEEKDAYS FEMALES
df_female_domsp_weekday <- df_domsp_weekday[df_domsp_weekday$Sex == 2,]
save(df_female_domsp_weekday, file = 'df_female_domsp_weekday.Rdata')

df_long <- df_female_domsp_weekday %>%
  distinct(hicid, .keep_all = TRUE) %>%
  pivot_longer(
    cols = ends_with(c("10", "12", "14")),
```

```
    names_to = c(".value", "time"),
    names_pattern = "(.*?)_(\\d\\d)"
  ) %>%
  mutate(across(where(is.numeric), ~ if_else(.x == 0, 0.01, .x)),
         time = as.integer(time)) %>%
  as.data.frame()

variables = c(
  "active_transport_at",
  "daytime_naps_at",
  "education_SB_at",
  "leisure_time_SB_at",
  "passive_transport_at",
  "screen_time_at",
  "self_care_SB_at",
  "social_based_SB_at",
  "structured_MVPA_at",
  "unstructured_LPA_at",
  "unstructured_MVPA_at",
  "household_LPA_at",
  "nighttime_sleep_at"
)

df_long_reduced <- df_long %>%
  select(variables, time, hicid)

# Setup the backend cluster
cl <- makeCluster(detectCores() - 1) # you can reduce this if needed
registerDoParallel(cl)

# Define the function to process
get_metrics <- function(df, variables) {
  foreach(ng = rep(2:6, 2), d = c(rep(1, 5), rep(2, 5))) %dopar% {
    tryCatch({
      set.seed(42)
      res <-
        gbmt::gbmt(
          x.names = variables,
          unit = "hicid",
          time = "time",
          d = d,
          ng = ng,
          data = df,
          nstart = 10,
```

```
        pruning = FALSE
      )
    res
  },
  error =  function(e)
    0)
 }
}

# Run the function
results_female_domsp_weekday <- get_metrics(df_long_reduced, variables)

# Stop the backend cluster
stopCluster(cl)

# Save results
save(results_female_domsp_weekday, file = 'results_female_domsp_weekday.Rdata')

# Put the results in a table
do.call(rbind, lapply(results_female_domsp_weekday, function(x) {
 y <- x$ic
 y["ng"] <- x$call$ng
 y["d"] <- x$call$d
 y
}))

# Make Universal CSV files for models 1-5 for FCAP analysis
make_csvs <- function(i) {
 grps <- results_female_domsp_weekday[[i]]$call$ng
 grpcols <- grps + 2
 first_line <-
  paste(
   results_female_domsp_weekday[[i]]$ic[["aic"]],
   results_female_domsp_weekday[[i]]$ic[["bic"]],
   paste0(results_female_domsp_weekday[[i]]$logLik, "\n"),
   sep = ","
  )
 out_table <-
  merge(results_female_domsp_weekday[[i]]$posterior,
      results_female_domsp_weekday[[i]]$assign,
      by = "row.names")[, 2:grpcols]
 filename <-
  paste0(
   "./FCAP/FCAP_female_domsp_weekday/FCAP CSV Files_models1_5/",
```

```
      "universal_",
      grps,
      ".csv"
    )
  cat(first_line, file = filename)
  write.table(
    out_table,
    file = filename,
    append = TRUE,
    sep = ",",
    row.names = FALSE,
    col.names = FALSE
  )
}

for (n in 1:5) {
  try{
    make_csvs(n)
  }
}

# Make Universal CSV files for models 6-10 for FCAP analysis
make_csvs <- function(i) {
  grps <- results_female_domsp_weekday[[i]]$call$ng
  grpcols <- grps + 2
  first_line <-
    paste(
      results_female_domsp_weekday[[i]]$ic[["aic"]],
      results_female_domsp_weekday[[i]]$ic[["bic"]],
      paste0(results_female_domsp_weekday[[i]]$logLik, "\n"),
      sep = ","
    )
  out_table <-
    merge(results_female_domsp_weekday[[i]]$posterior,
          results_female_domsp_weekday[[i]]$assign,
          by = "row.names")[, 2:grpcols]
  filename <-
    paste0(
      "./FCAP/FCAP_female_domsp_weekday/FCAP CSV Files_models6_10/",
      "universal_",
      grps,
      ".csv"
    )
  cat(first_line, file = filename)
```

```
  write.table(
    out_table,
    file = filename,
    append = TRUE,
    sep = ",",
    row.names = FALSE,
    col.names = FALSE
  )
}

for (n in 6:10) {
  try{
    make_csvs(n)
  }
}



# GENERAL WEEKDAYS SENSITIVITY ANALYSIS
df <- read_dta(file = "Z:/LSAC
dataset/Study_2/Study_2/Multi_Trajecotry_Analysis_Domain_Specific_Movement_Behaviours.d
ta")

df_sens_gen_weekday <- df[df$day_of_week_at_10 == 1,]
save(df_sens_gen_weekday, file = 'df_sens_gen_weekday.Rdata')

#MALE SENSITIVITY GENERAL WEEKDAY

df_sens_male_gen_weekday <- df_sens_gen_weekday[df_sens_gen_weekday$Sex == 1,]
save(df_sens_male_gen_weekday, file = 'df_sens_male_gen_weekday.Rdata')

df_long <- df_sens_male_gen_weekday %>%
  distinct(hicid, .keep_all = TRUE) %>%
  pivot_longer(
    cols = ends_with(c("10", "12", "14")),
    names_to = c(".value", "time"),
    names_pattern = "(.*?)_(\\d\\d)"
  ) %>%
  mutate(across(where(is.numeric), ~ if_else(.x == 0, 0.01, .x)),
       time = as.integer(time)) %>%
  as.data.frame()

variables = c("LPA_at", "MVPA_at", "SB_at", "sleep_at")
```

```
df_long_reduced <- df_long %>%
  select(variables, time, hicid)


# Setup the backend cluster
cl <- makeCluster(detectCores() - 1) # you can reduce this if needed
registerDoParallel(cl)

# Define the function to process
get_metrics <- function(df, variables) {
  foreach(ng = rep(2:6, 2), d = c(rep(1, 5), rep(2, 5))) %dopar% {
    tryCatch({
      set.seed(42)
      res <-
        gbmt::gbmt(
          x.names = variables,
          unit = "hicid",
          time = "time",
          d = d,
          ng = ng,
          data = df,
          nstart = 10,
          pruning = FALSE
        )
      res
    },
    error =  function(e)
      0)
  }
}

# Run the function
results_sens_male_gen_weekday <- get_metrics(df_long_reduced, variables)

stopCluster(cl)

# Save results
save(results_sens_male_gen_weekday, file = 'results_sens_male_gen_weekday.Rdata')

# Put the results in a table
do.call(rbind, lapply(results_sens_male_gen_weekday, function(x) {
  y <- x$ic
  y["ng"] <- x$call$ng
  y["d"] <- x$call$d
```

```
  y
}))

# Make Universal CSV files for models 1-5 for FCAP analysis
make_csvs <- function(i) {
  grps <- results_sens_male_gen_weekday[[i]]$call$ng
  grpcols <- grps + 2
  first_line <-
    paste(
      results_sens_male_gen_weekday[[i]]$ic[["aic"]],
      results_sens_male_gen_weekday[[i]]$ic[["bic"]],
      paste0(results_sens_male_gen_weekday[[i]]$logLik, "\n"),
      sep = ","
    )
  out_table <-
    merge(results_sens_male_gen_weekday[[i]]$posterior,
          results_sens_male_gen_weekday[[i]]$assign,
          by = "row.names")[, 2:grpcols]
  filename <-
    paste0(
      "./FCAP/Sensitivity Analyses/FCAP_male_gen_weekdays_sensitivity/FCAP CSV
Files_models1_5/",
      "universal_",
      grps,
      ".csv"
    )
  cat(first_line, file = filename)
  write.table(
    out_table,
    file = filename,
    append = TRUE,
    sep = ",",
    row.names = FALSE,
    col.names = FALSE
  )
}

for (n in 1:5) {
  try(
    make_csvs(n)
  )
}

# Make Universal CSV files for models 6-10 for FCAP analysis
```

```
make_csvs <- function(i) {
  grps <- results_sens_male_gen_weekday[[i]]$call$ng
  grpcols <- grps + 2
  first_line <-
    paste(
      results_sens_male_gen_weekday[[i]]$ic[["aic"]],
      results_sens_male_gen_weekday[[i]]$ic[["bic"]],
      paste0(results_sens_male_gen_weekday[[i]]$logLik, "\n"),
      sep = ","
    )
  out_table <-
    merge(results_sens_male_gen_weekday[[i]]$posterior,
          results_sens_male_gen_weekday[[i]]$assign,
          by = "row.names")[, 2:grpcols]
  filename <-
    paste0(
      "./FCAP/Sensitivity Analyses/FCAP_male_gen_weekdays_sensitivity/FCAP CSV
Files_models6_10/",
      "universal_",
      grps,
      ".csv"
    )
  cat(first_line, file = filename)
  write.table(
    out_table,
    file = filename,
    append = TRUE,
    sep = ",",
    row.names = FALSE,
    col.names = FALSE
  )
}

for (n in 6:10) {
  try(
    make_csvs(n)
  )
}

#FEMALE GENERAL WEEKDAY SENSITIVITY
df_sens_female_gen_weekday <- df_sens_gen_weekday[df_sens_gen_weekday$Sex == 2,]
save(df_sens_female_gen_weekday, file = 'df_sens_female_gen_weekday.Rdata')

df_long <- df_sens_female_gen_weekday %>%
```

```
  distinct(hicid, .keep_all = TRUE) %>%
  pivot_longer(
    cols = ends_with(c("10", "12", "14")),
    names_to = c(".value", "time"),
    names_pattern = "(.*?)_(\\d\\d)"
  ) %>%
  mutate(across(where(is.numeric), ~ if_else(.x == 0, 0.01, .x)),
         time = as.integer(time)) %>%
  as.data.frame()

variables = c("LPA_at", "MVPA_at", "SB_at", "sleep_at")

df_long_reduced <- df_long %>%
  select(variables, time, hicid)


# Setup the backend cluster
cl <- makeCluster(detectCores() - 1) # you can reduce this if needed
registerDoParallel(cl)

# Define the function to process
get_metrics <- function(df, variables) {
  foreach(ng = rep(2:6, 2), d = c(rep(1, 5), rep(2, 5))) %dopar% {
    tryCatch({
      set.seed(42)
      res <-
        gbmt::gbmt(
          x.names = variables,
          unit = "hicid",
          time = "time",
          d = d,
          ng = ng,
          data = df,
          nstart = 10,
          pruning = FALSE
        )
      res
    },
    error =  function(e)
      0)
  }
}

# Run the function
```

```
results_sens_female_gen_weekday <- get_metrics(df_long_reduced, variables)

stopCluster(cl)

# Save results
save(results_sens_female_gen_weekday, file = 'results_sens_female_gen_weekday.Rdata')

# Put the results in a table
do.call(rbind, lapply(results_sens_female_gen_weekday, function(x) {
  y <- x$ic
  y["ng"] <- x$call$ng
  y["d"] <- x$call$d
  y
}))

# Make Universal CSV files for models 1-5 for FCAP analysis
make_csvs <- function(i) {
  grps <- results_sens_female_gen_weekday[[i]]$call$ng
  grpcols <- grps + 2
  first_line <-
    paste(
      results_sens_female_gen_weekday[[i]]$ic[["aic"]],
      results_sens_female_gen_weekday[[i]]$ic[["bic"]],
      paste0(results_sens_female_gen_weekday[[i]]$logLik, "\n"),
      sep = ","
    )
  out_table <-
    merge(results_sens_female_gen_weekday[[i]]$posterior,
          results_sens_female_gen_weekday[[i]]$assign,
          by = "row.names")[, 2:grpcols]
  filename <-
    paste0(
      "./FCAP/Sensitivity Analyses/FCAP_female_gen_weekdays_sensitivity/FCAP CSV
Files_models1_5/",
      "universal_",
      grps,
      ".csv"
    )
  cat(first_line, file = filename)
  write.table(
    out_table,
    file = filename,
    append = TRUE,
    sep = ",",
```

```
    row.names = FALSE,
    col.names = FALSE
  )
}

for (n in 1:5) {
  try(
    make_csvs(n)
  )
}

# Make Universal CSV files for models 6-10 for FCAP analysis
make_csvs <- function(i) {
  grps <- results_sens_female_gen_weekday[[i]]$call$ng
  grpcols <- grps + 2
  first_line <-
    paste(
      results_sens_female_gen_weekday[[i]]$ic[["aic"]],
      results_sens_female_gen_weekday[[i]]$ic[["bic"]],
      paste0(results_sens_female_gen_weekday[[i]]$logLik, "\n"),
      sep = ","
    )
  out_table <-
    merge(results_sens_female_gen_weekday[[i]]$posterior,
          results_sens_female_gen_weekday[[i]]$assign,
          by = "row.names")[, 2:grpcols]
  filename <-
    paste0(
      "./FCAP/Sensitivity Analyses/FCAP_female_gen_weekdays_sensitivity/FCAP CSV
Files_models6_10/",
      "universal_",
      grps,
      ".csv"
    )
  cat(first_line, file = filename)
  write.table(
    out_table,
    file = filename,
    append = TRUE,
    sep = ",",
    row.names = FALSE,
    col.names = FALSE
  )
}
```

```
for (n in 6:10) {
  try(
    make_csvs(n)
  )
}

# DOMAIN-SPECIFIC WEEKDAYS SENSITIVITY
df_sens_domsp_weekday <- df[df$day_of_week_at_10 == 1,]
save(df_sens_domsp_weekday, file = 'df_sens_domsp_weekday.Rdata')

#MALE DOMAIN-SPECIFIC WEEKDAY SENSITIVITY
df_sens_male_domsp_weekday <- df_sens_domsp_weekday[df_sens_domsp_weekday$Sex
== 1,]
save(df_sens_male_domsp_weekday, file = 'df_sens_male_domsp_weekday.Rdata')

df_long <- df_sens_male_domsp_weekday %>%
  distinct(hicid, .keep_all = TRUE) %>%
  pivot_longer(
    cols = ends_with(c("10", "12", "14")),
    names_to = c(".value", "time"),
    names_pattern = "(.*?)_(\\d\\d)"
  ) %>%
  mutate(across(where(is.numeric), ~ if_else(.x == 0, 0.01, .x)),
       time = as.integer(time)) %>%
  as.data.frame()

variables = c(
  "active_transport_at",
  "daytime_naps_at",
  "education_SB_at",
  "leisure_time_SB_at",
  "passive_transport_at",
  "screen_time_at",
  "self_care_SB_at",
  "social_based_SB_at",
  "structured_MVPA_at",
  "unstructured_LPA_at",
  "unstructured_MVPA_at",
  "household_LPA_at",
  "nighttime_sleep_at"
)

df_long_reduced <- df_long %>%
```

```
  select(variables, time, hicid)

# Setup the backend cluster
cl <- makeCluster(detectCores() - 1) # you can reduce this if needed
registerDoParallel(cl)

# Define the function to process
get_metrics <- function(df, variables) {
  foreach(ng = rep(2:6, 2), d = c(rep(1, 5), rep(2, 5))) %dopar% {
    tryCatch({
      set.seed(42)
      res <-
        gbmt::gbmt(
          x.names = variables,
          unit = "hicid",
          time = "time",
          d = d,
          ng = ng,
          data = df,
          nstart = 10,
          pruning = FALSE
        )
      res
    },
    error =  function(e)
      0)
  }
}
# Run the function
results_sens_male_domsp_weekday <-
  get_metrics(df_long_reduced, variables)

# Stop the backend cluster
stopCluster(cl)

#Save results
save(results_sens_male_domsp_weekday, file = 'results_sens_male_domsp_weekday.Rdata')

# Put the results in a table
do.call(rbind, lapply(results_sens_male_domsp_weekday, function(x) {
  y <- x$ic
  y["ng"] <- x$call$ng
  y["d"] <- x$call$d
  y
```

```
}))

# Make Universal CSV files for models 1-5 for FCAP analysis
make_csvs <- function(i) {
  grps <- results_sens_male_domsp_weekday[[i]]$call$ng
  grpcols <- grps + 2
  first_line <-
    paste(
      results_sens_male_domsp_weekday[[i]]$ic[["aic"]],
      results_sens_male_domsp_weekday[[i]]$ic[["bic"]],
      paste0(results_sens_male_domsp_weekday[[i]]$logLik, "\n"),
      sep = ","
    )
  out_table <-
    merge(results_sens_male_domsp_weekday[[i]]$posterior,
          results_sens_male_domsp_weekday[[i]]$assign,
          by = "row.names")[, 2:grpcols]
  filename <-
    paste0(
      "./FCAP/Sensitivity Analyses/FCAP_male_domsp_weekdays_sensitivity/FCAP CSV
Files_models1_5/",
      "universal_",
      grps,
      ".csv"
    )
  cat(first_line, file = filename)
  write.table(
    out_table,
    file = filename,
    append = TRUE,
    sep = ",",
    row.names = FALSE,
    col.names = FALSE
  )
}

for (n in 1:5) {
  try{
    make_csvs(n)
  }
}

# Make Universal CSV files for models 6-10 for FCAP analysis
make_csvs <- function(i) {
```

```
  grps <- results_sens_male_domsp_weekday[[i]]$call$ng
  grpcols <- grps + 2
  first_line <-
    paste(
      results_sens_male_domsp_weekday[[i]]$ic[["aic"]],
      results_sens_male_domsp_weekday[[i]]$ic[["bic"]],
      paste0(results_sens_male_domsp_weekday[[i]]$logLik, "\n"),
      sep = ","
    )
  out_table <-
    merge(results_sens_male_domsp_weekday[[i]]$posterior,
          results_sens_male_domsp_weekday[[i]]$assign,
          by = "row.names")[, 2:grpcols]
  filename <-
    paste0(
      "./FCAP/Sensitivity Analyses/FCAP_male_domsp_weekdays_sensitivity/FCAP CSV
Files_models6_10/",
      "universal_",
      grps,
      ".csv"
    )
  cat(first_line, file = filename)
  write.table(
    out_table,
    file = filename,
    append = TRUE,
    sep = ",",
    row.names = FALSE,
    col.names = FALSE
  )
}

for (n in 6:10) {
  try{
    make_csvs(n)
  }
}

#female DOMAIN-SPECIFIC WEEKDAY SENSITIVITY
df_sens_female_domsp_weekday <- df_sens_domsp_weekday[df_sens_domsp_weekday$Sex
== 2,]
save(df_sens_female_domsp_weekday, file = 'df_sens_female_domsp_weekday.Rdata')

df_long <- df_sens_female_domsp_weekday %>%
```

```
  distinct(hicid, .keep_all = TRUE) %>%
  pivot_longer(
    cols = ends_with(c("10", "12", "14")),
    names_to = c(".value", "time"),
    names_pattern = "(.*?)_(\\d\\d)"
  ) %>%
  mutate(across(where(is.numeric), ~ if_else(.x == 0, 0.01, .x)),
         time = as.integer(time)) %>%
  as.data.frame()

variables = c(
  "active_transport_at",
  "daytime_naps_at",
  "education_SB_at",
  "leisure_time_SB_at",
  "passive_transport_at",
  "screen_time_at",
  "self_care_SB_at",
  "social_based_SB_at",
  "structured_MVPA_at",
  "unstructured_LPA_at",
  "unstructured_MVPA_at",
  "household_LPA_at",
  "nighttime_sleep_at"
)

df_long_reduced <- df_long %>%
  select(variables, time, hicid)

# Setup the backend cluster
cl <- makeCluster(detectCores() - 1) # you can reduce this if needed
registerDoParallel(cl)

# Define the function to process
get_metrics <- function(df, variables) {
  foreach(ng = rep(2:6, 2), d = c(rep(1, 5), rep(2, 5))) %dopar% {
    tryCatch({
      set.seed(42)
      res <-
        gbmt::gbmt(
          x.names = variables,
          unit = "hicid",
          time = "time",
          d = d,
```

```
          ng = ng,
          data = df,
          nstart = 10,
          pruning = FALSE
        )
      res
    },
    error =  function(e)
      0)
  }
}
# Run the function
results_sens_female_domsp_weekday <-
  get_metrics(df_long_reduced, variables)

# Stop the backend cluster
stopCluster(cl)

#Save results
save(results_sens_female_domsp_weekday, file =
'results_sens_female_domsp_weekday.Rdata')

# Put the results in a table
do.call(rbind, lapply(results_sens_female_domsp_weekday, function(x) {
  y <- x$ic
  y["ng"] <- x$call$ng
  y["d"] <- x$call$d
  y
}))

# Make Universal CSV files for models 1-5 for FCAP analysis
make_csvs <- function(i) {
  grps <- results_sens_female_domsp_weekday[[i]]$call$ng
  grpcols <- grps + 2
  first_line <-
    paste(
      results_sens_female_domsp_weekday[[i]]$ic[["aic"]],
      results_sens_female_domsp_weekday[[i]]$ic[["bic"]],
      paste0(results_sens_female_domsp_weekday[[i]]$logLik, "\n"),
      sep = ","
    )
  out_table <-
    merge(results_sens_female_domsp_weekday[[i]]$posterior,
          results_sens_female_domsp_weekday[[i]]$assign,
```

```
          by = "row.names")[, 2:grpcols]
  filename <-
    paste0(
      "./FCAP/Sensitivity Analyses/FCAP_female_domsp_weekdays_sensitivity/FCAP CSV
Files_models1_5/",
      "universal_",
      grps,
      ".csv"
    )
  cat(first_line, file = filename)
  write.table(
    out_table,
    file = filename,
    append = TRUE,
    sep = ",",
    row.names = FALSE,
    col.names = FALSE
  )
}

for (n in 1:5) {
  try{
    make_csvs(n)
  }
}

# Make Universal CSV files for models 6-10 for FCAP analysis
make_csvs <- function(i) {
  grps <- results_sens_female_domsp_weekday[[i]]$call$ng
  grpcols <- grps + 2
  first_line <-
    paste(
      results_sens_female_domsp_weekday[[i]]$ic[["aic"]],
      results_sens_female_domsp_weekday[[i]]$ic[["bic"]],
      paste0(results_sens_female_domsp_weekday[[i]]$logLik, "\n"),
      sep = ","
    )
  out_table <-
    merge(results_sens_female_domsp_weekday[[i]]$posterior,
          results_sens_female_domsp_weekday[[i]]$assign,
          by = "row.names")[, 2:grpcols]
  filename <-
    paste0(
```

```
      "./FCAP/Sensitivity Analyses/FCAP_female_domsp_weekdays_sensitivity/FCAP CSV
Files_models6_10/",
      "universal_",
      grps,
      ".csv"
    )
  cat(first_line, file = filename)
  write.table(
    out_table,
    file = filename,
    append = TRUE,
    sep = ",",
    row.names = FALSE,
    col.names = FALSE
  )
}

for (n in 6:10) {
  try{
    make_csvs(n)
  }
}
```
