# Supplementary material for: Socioeconomic position as a predictor of youth's movement trajectory profiles between ages 10 and 14 years
Source: Int J Behav Nutr Phys Act. 2023 Jul 22;20:88. doi: 10.1186/s12966-023-01491-5 (PMC10363305; doi:10.1186/s12966-023-01491-5)
Supplement: Supplementary file 4 — Additional file 4. Fit-criteria assessment plots for choosing general and domain-specific models. [file 12966_2023_1491_MOESM4_ESM.pdf]

Additional file S3.

Fit-Criteria Assessment Plots (General Male)

Linear Polynomial

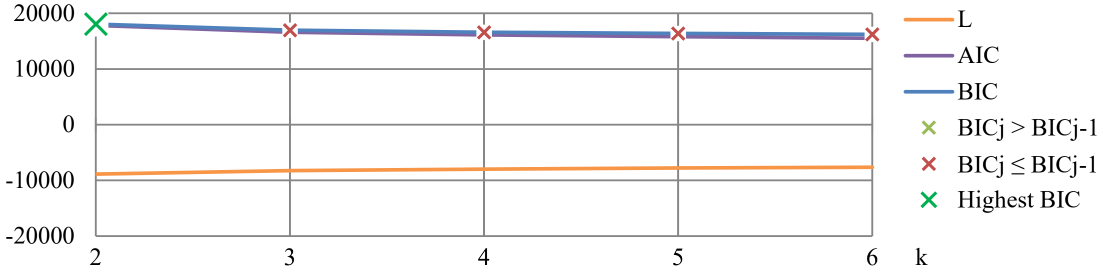

Akaike's information criterion, Bayesian information criterion and Likelihood

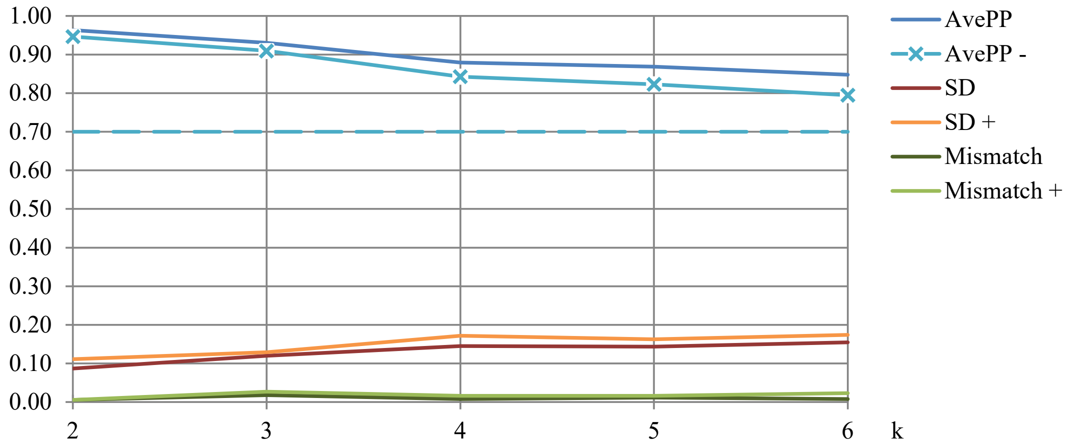

Posterior probability, mismatch and SD

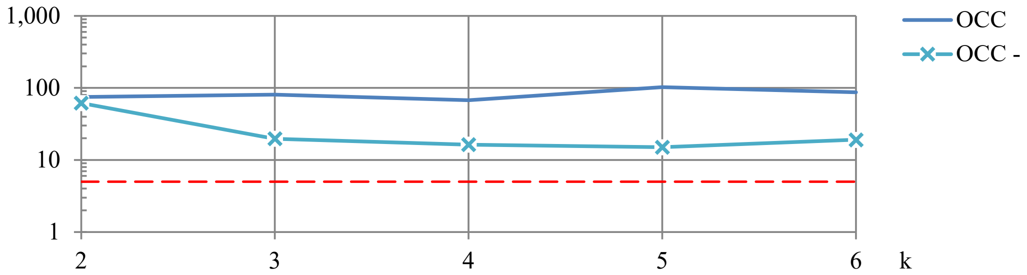

Odds of correct classification

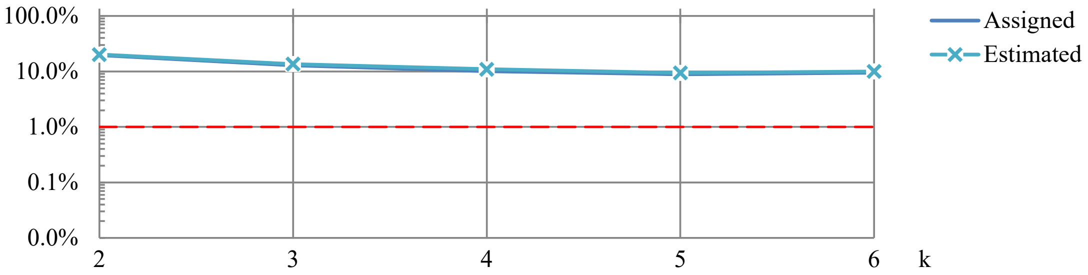

Percentage of individuals belonging to the smallest group

Fit-Criteria Assessment Plots (General Male)

Quadratic Polynomial

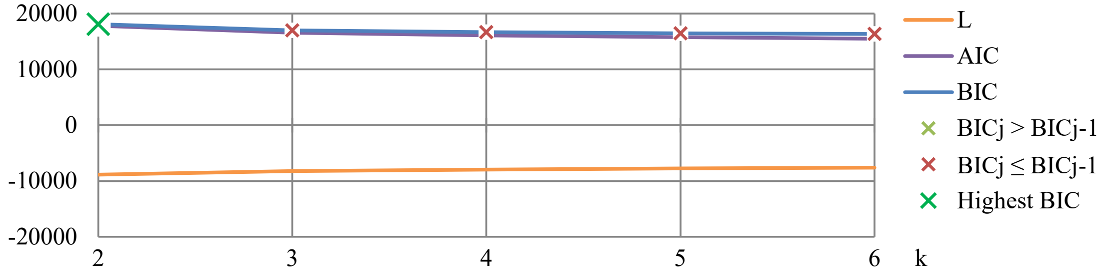

Akaike's information criterion, Bayesian information criterion and Likelihood

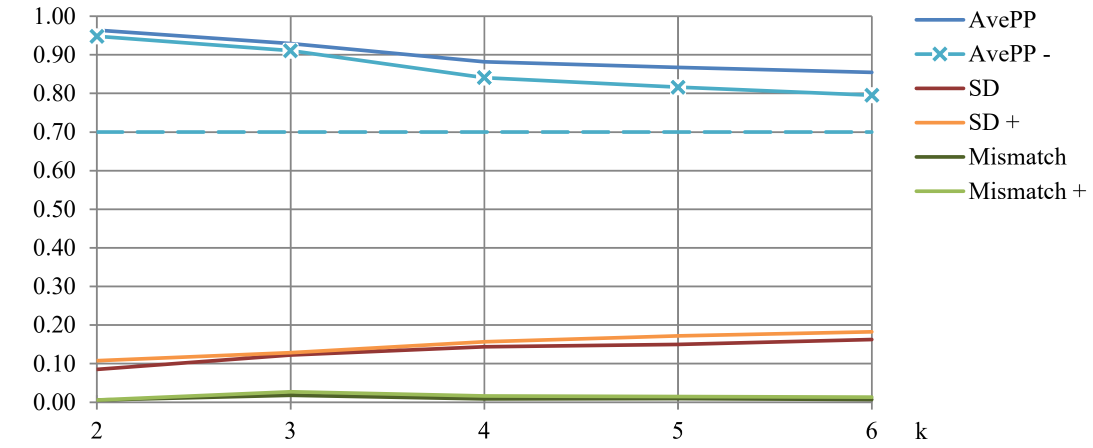

Posterior probability, mismatch and SD

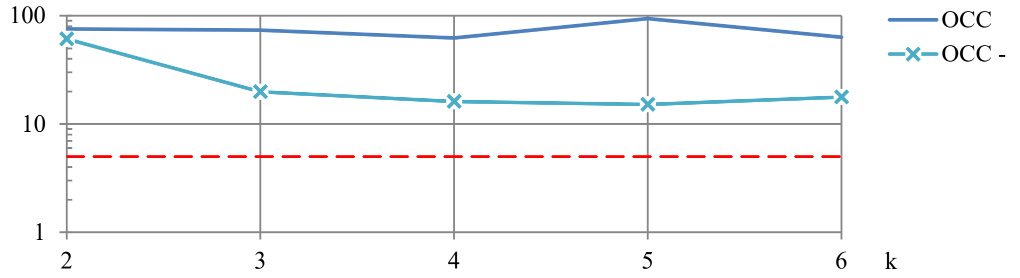

Odds of correct classification

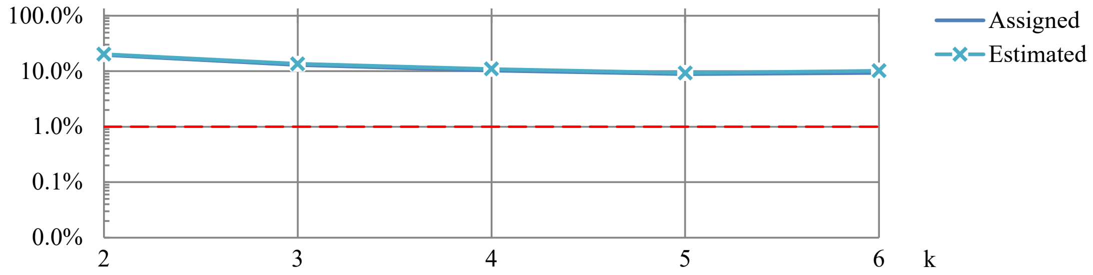

Percentage of individuals belonging to the smallest group

Fit-Criteria Assessment Plots (General Female)

Linear Polynomial

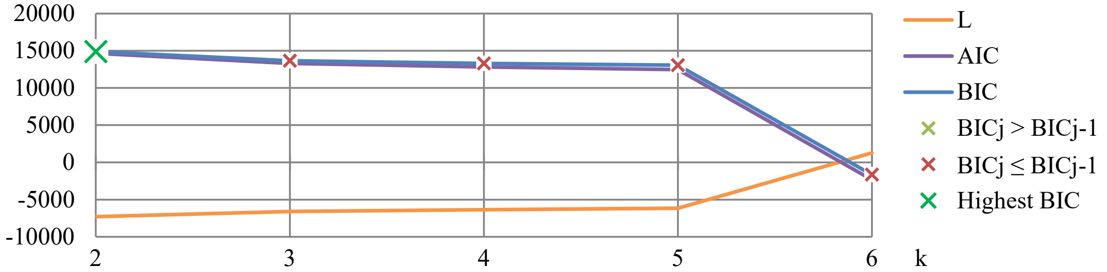

Akaike's information criterion, Bayesian information criterion and Likelihood

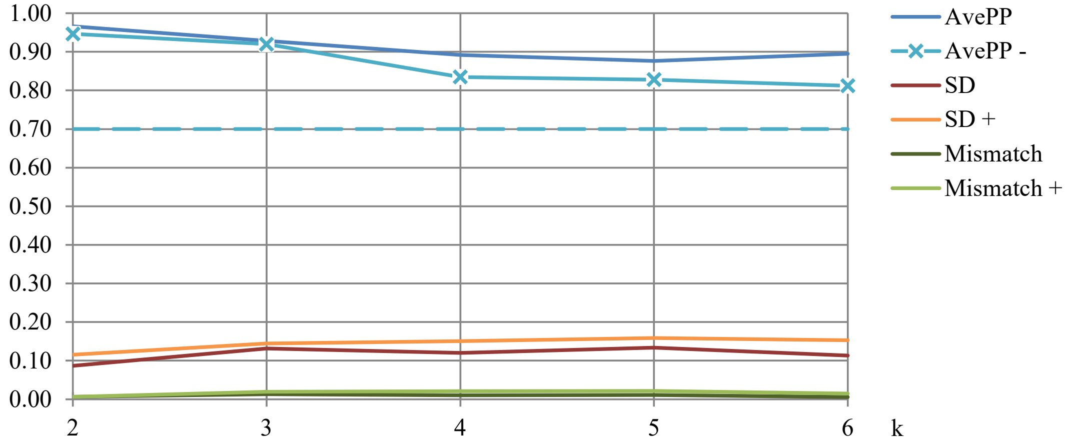

Posterior probability, mismatch and SD

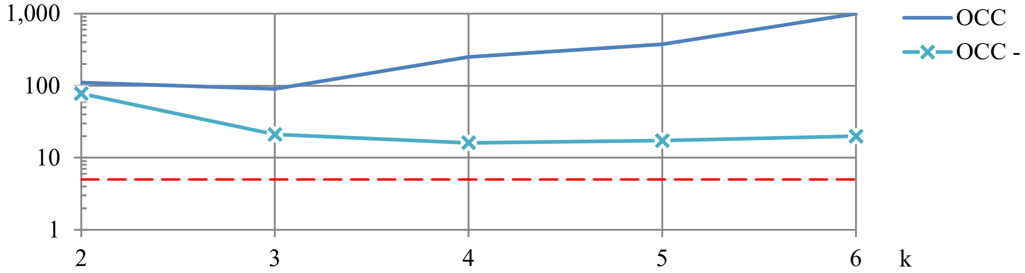

Odds of correct classification

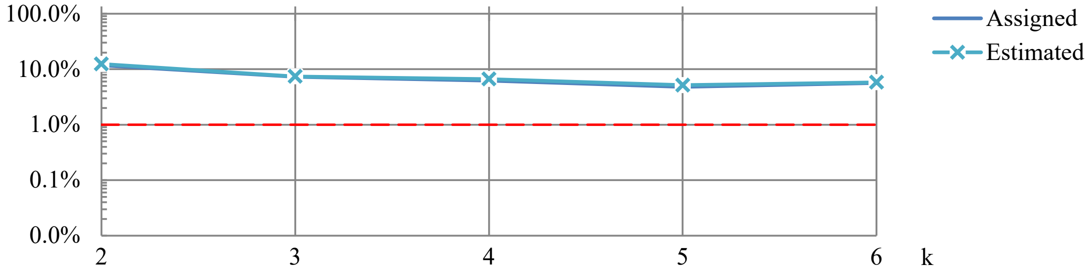

Percentage of individuals belonging to the smallest group

Fit-Criteria Assessment Plots (General Female)

Quadratic Polynomial

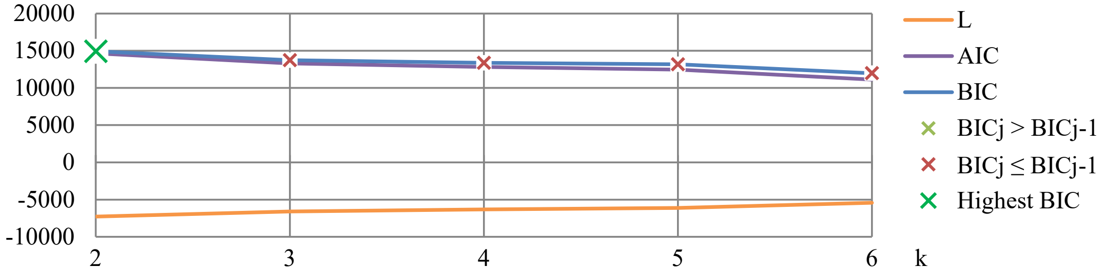

Akaike's information criterion, Bayesian information criterion and Likelihood

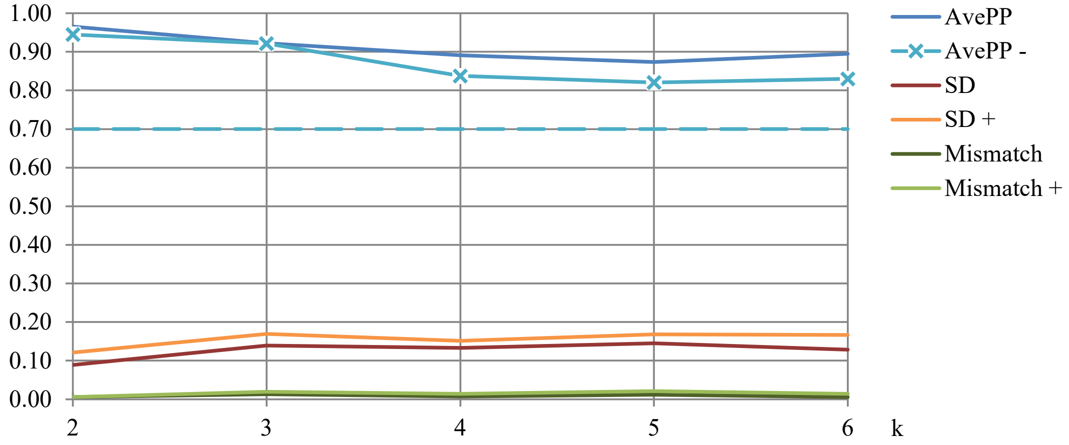

Posterior probability, mismatch and SD

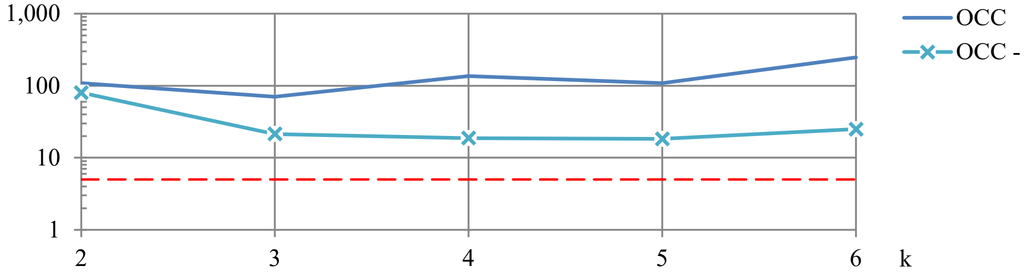

Odds of correct classification

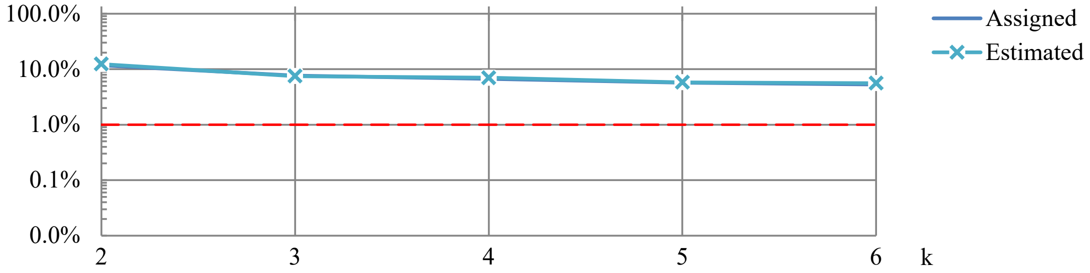

Percentage of individuals belonging to the smallest group

Fit-Criteria Assessment Plots (Domain-specific Male)

Linear Polynomial

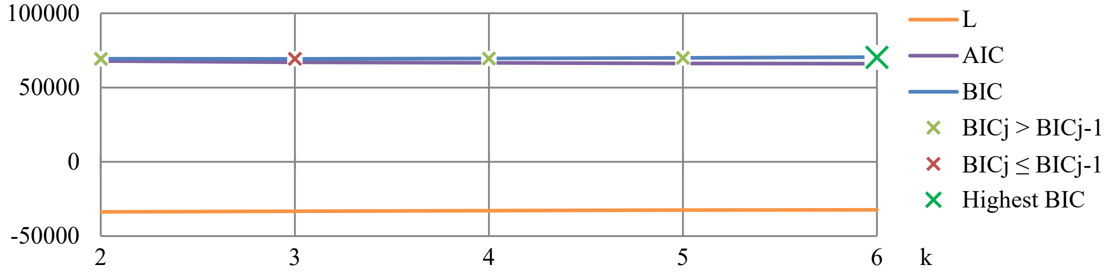

Akaike's information criterion, Bayesian information criterion and Likelihood

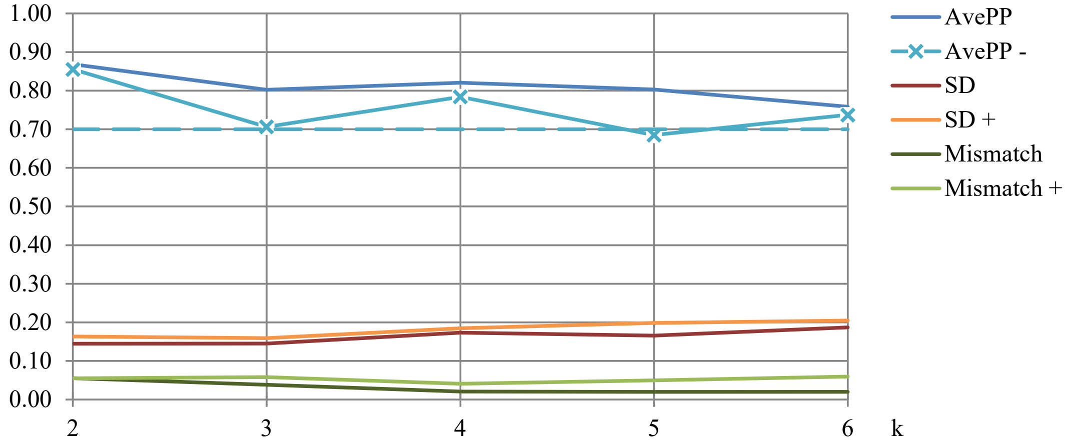

Posterior probability, mismatch and SD

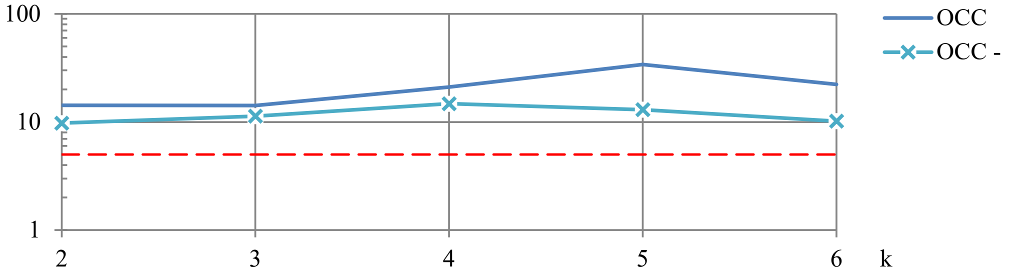

Odds of correct classification

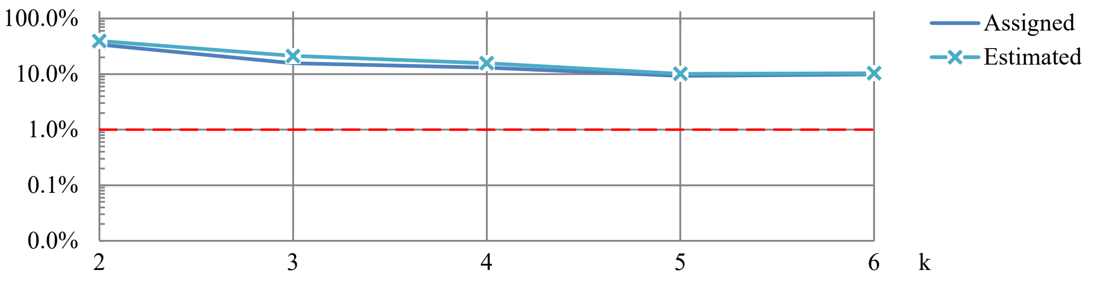

Percentage of individuals belonging to the smallest group

Fit-Criteria Assessment Plots (Domain-specific Male)

Quadratic Polynomial

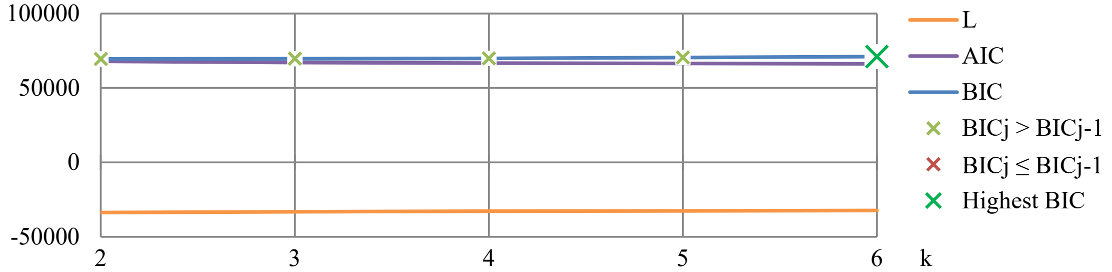

Akaike's information criterion, Bayesian information criterion and Likelihood

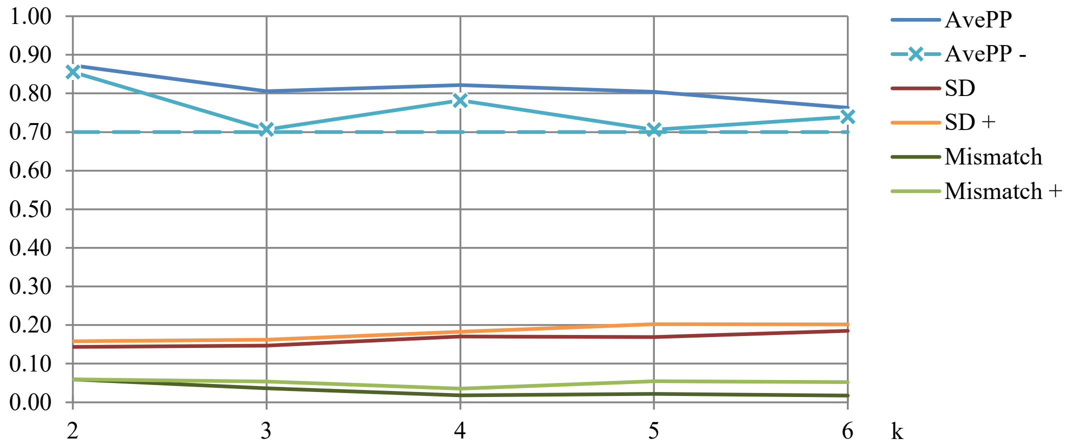

Posterior probability, mismatch and SD

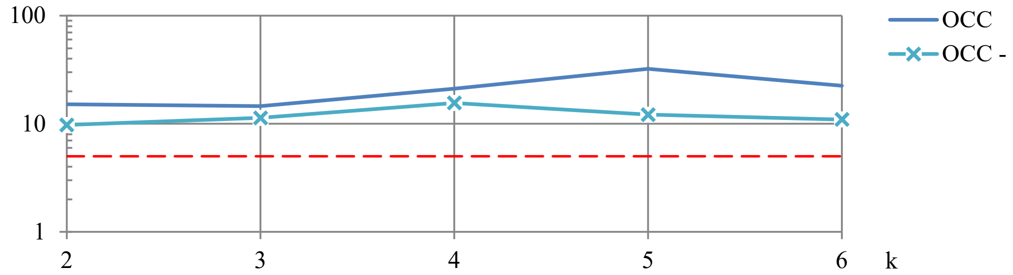

Odds of correct classification

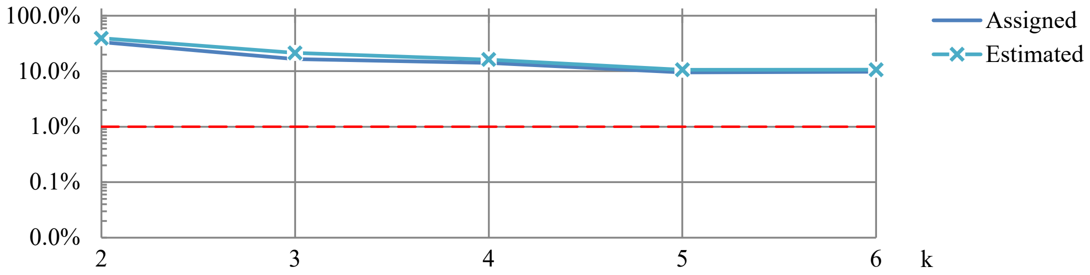

Percentage of individuals belonging to the smallest group

Fit-Criteria Assessment Plots (Domain-specific Female)

Linear polynomial

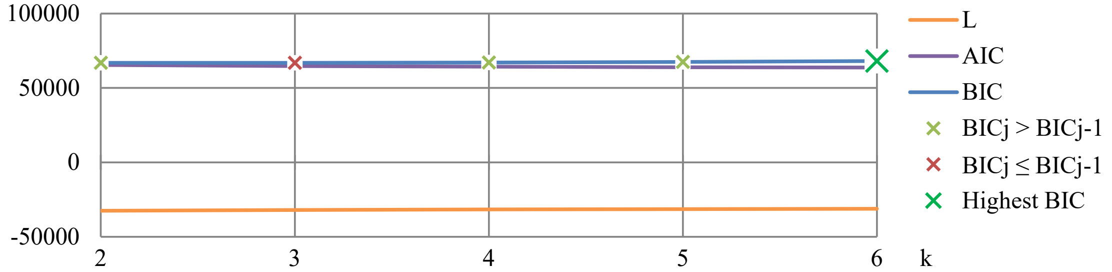

Akaike's information criterion, Bayesian information criterion and Likelihood

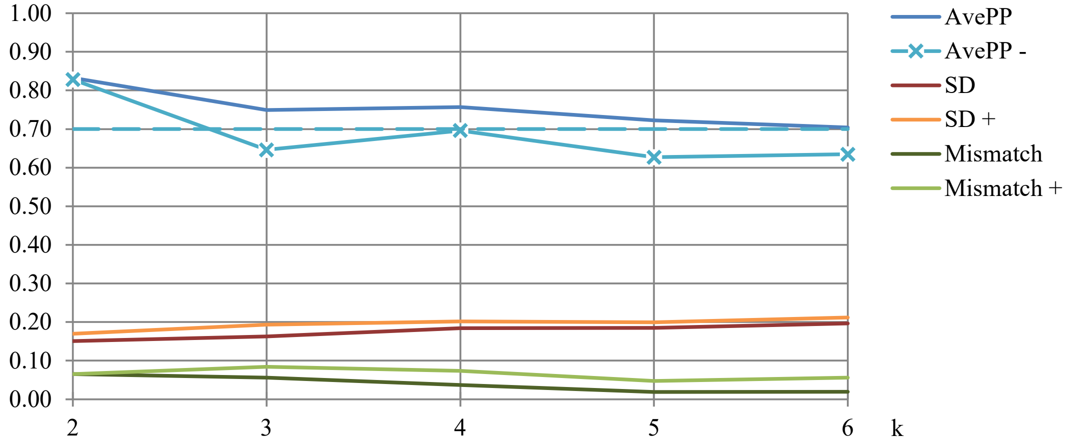

Posterior probability, mismatch and SD

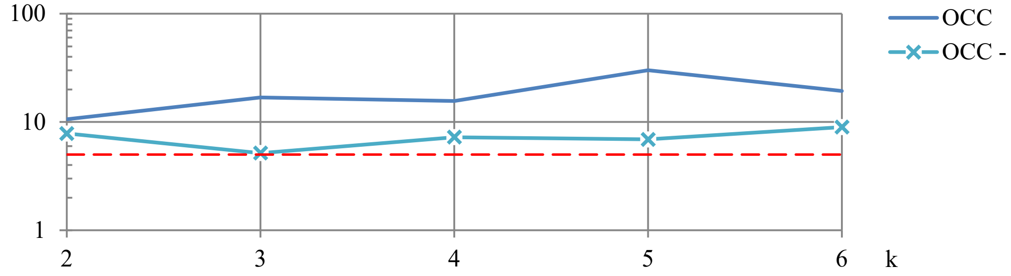

Odds of correct classification

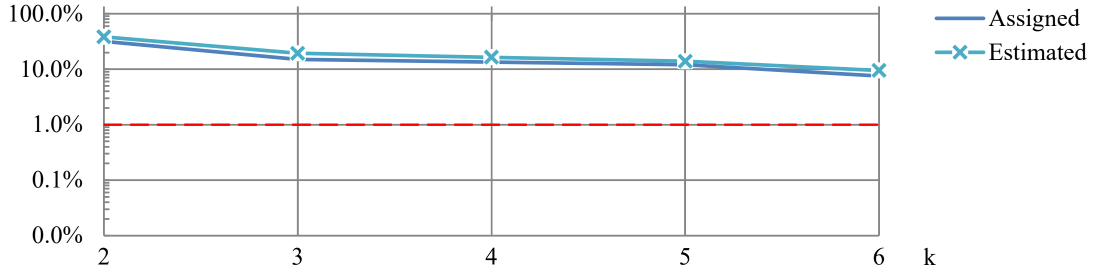

Percentage of individuals belonging to the smallest group

Fit-Criteria Assessment Plots (Domain-specific Female)

Quadratic Polynomial

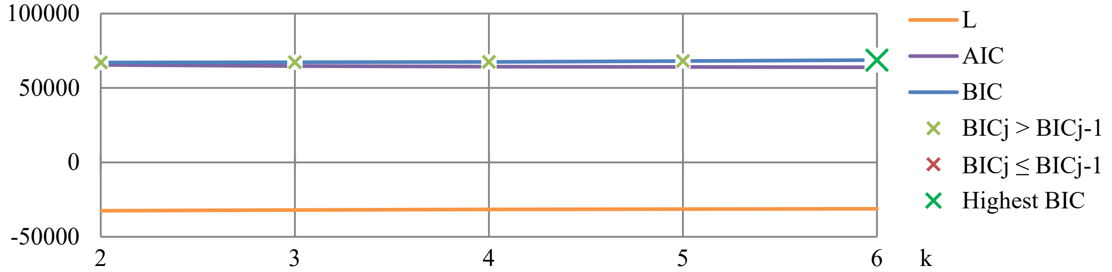

Akaike's information criterion, Bayesian information criterion and Likelihood

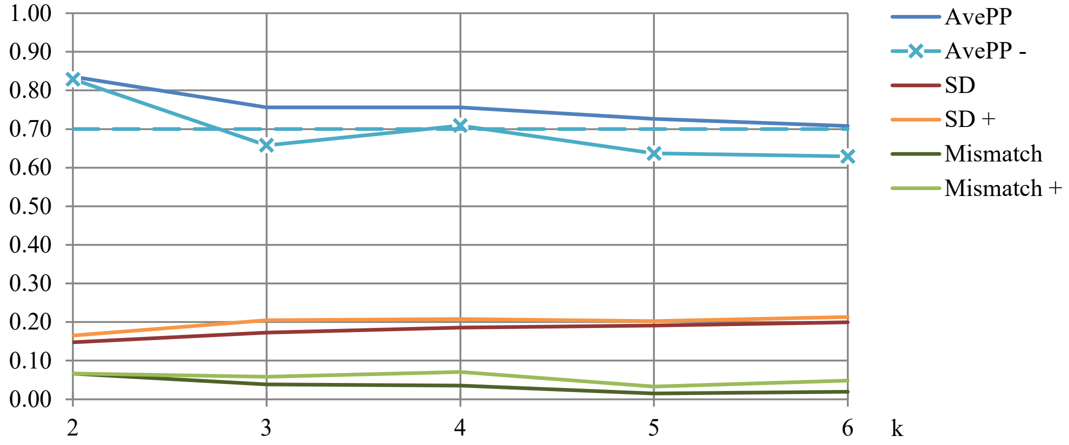

Posterior probability, mismatch and SD

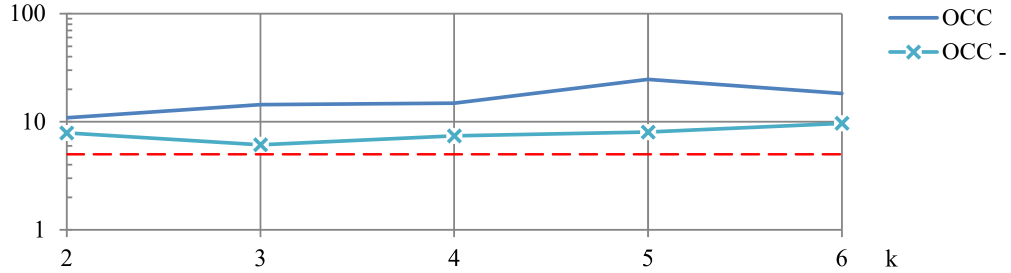

Odds of correct classification

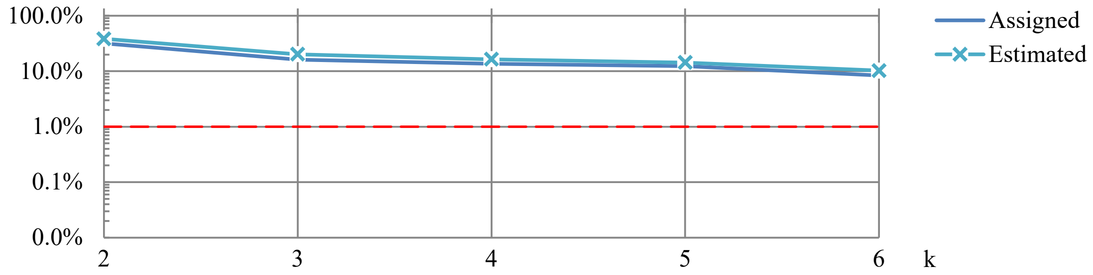

Percentage of individuals belonging to the smallest group
